# Odd‐even effects in molecular junctions: A Solomonic resolution of conflicting reports on conductance trends, metal specificity, and microscopic origin

**DOI:** 10.1002/smo2.70108

**Published:** 2026-10-08

**Authors:** Jiajun Feng, Ioan Bâldea, Zuoti Xie

**Affiliations:** ^1^ Guangdong Provincial Key Laboratory of Materials and Technologies for Energy Conversion, Department of Materials Science and Engineering Guangdong Technion‐Israel Institute of Technology Shantou Guangdong China; ^2^ Schulich Faculty of Chemistry Technion–Israel Institute of Technology Haifa Israel; ^3^ Theoretical Chemistry Heidelberg University Heidelberg Germany; ^4^ Quantum Science Center of Guangdong‐Hong Kong‐Macao Greater Bay Area Shenzhen‐Hong Kong International Science and Technology Park Shenzhen Guangdong China

**Keywords:** molecular electronics, molecular junctions, nanotransport, odd‐even effects

## Abstract

Odd–even conductance alternation in alkanethiol (CnT, n=7–12) self‐assembled monolayers has been debated for two decades, with conflicting reports on trends, metal dependence, and microscopic origin. Using conducting probe atomic force microscopy (CP‐AFM) with Ag, Au, and Pt electrodes, we extract low‐bias conductance G and effective molecule–electrode coupling Γ via the off‐resonant single‐level model, combined with rigorous z‐score statistics (Welch's t‐test for unequal variances). We present a Solomonic resolution—meaning a balanced compromise that recognizes truth on both sides—to these long‐standing controversies. First, the odd–even effect has a dual origin, manifesting in both the contact conductance prefactor ($G_{c}$, $\Gamma_{c}$) and the length‐decay constant (β, $\bar{\beta }$). Interface and bulk contributions are therefore not mutually exclusive. The assignment to interface versus bulk remains inferential from transport parameters; independent structural characterization would be desirable but faces substantial experimental challenges. Second, the relative conductance of odd versus even chains reverses with length: odd chains are more conductive at short lengths, while even chains become superior beyond a crossover. A point estimate near $n_{c}\;\approx \;12$–13 emerges from the global fits, yet the limited experimental window (n=7–12) and the small difference in decay constants produce a wide uncertainty interval; the precise location of the crossover therefore remains undetermined. The qualitative inversion itself (from $G_{o}>G_{e}$ to $G_{e}>G_{o}$), however, is a robust finding and offers a natural reconciliation of prior opposing claims. Third, the effect is observed in CP‐AFM junctions under the present experimental conditions. Earlier CP‐AFM analyses of CnT junctions did not report it because they employed parity‐agnostic fitting; once the data are deliberately split by parity the effect becomes statistically clear. Fourth, metal dependence follows parity‐insensitive work‐function scaling G∝exp(ξΦ) with $\xi \;\approx \;3.1\;{\text{eV}}^{-1}$. Paradoxically, although absolute odd–even differences are smallest on Ag (conductances ∼20 times lower than on Au), statistical confidence is highest for Ag (>99.999 %), owing to exceptional reproducibility of even‐chain data. This analysis demonstrates that “weaker” does not imply “absent” and highlights the necessity of variance‐aware statistics. Apparently contradictory literature findings arise from different regimes of a single underlying phenomenon.

## Graphical abstract

By combining extensive transport data with the off‐resonant single‐level model and z‐score statistics, we demonstrate that the odd–even alternation is platform‐independent, of dual interface–bulk origin, and exhibits a conductance crossover at *n* = 12—reconciling contradictory literature and providing a Solomonic solution to debates on trends, metal specificity, and origin.

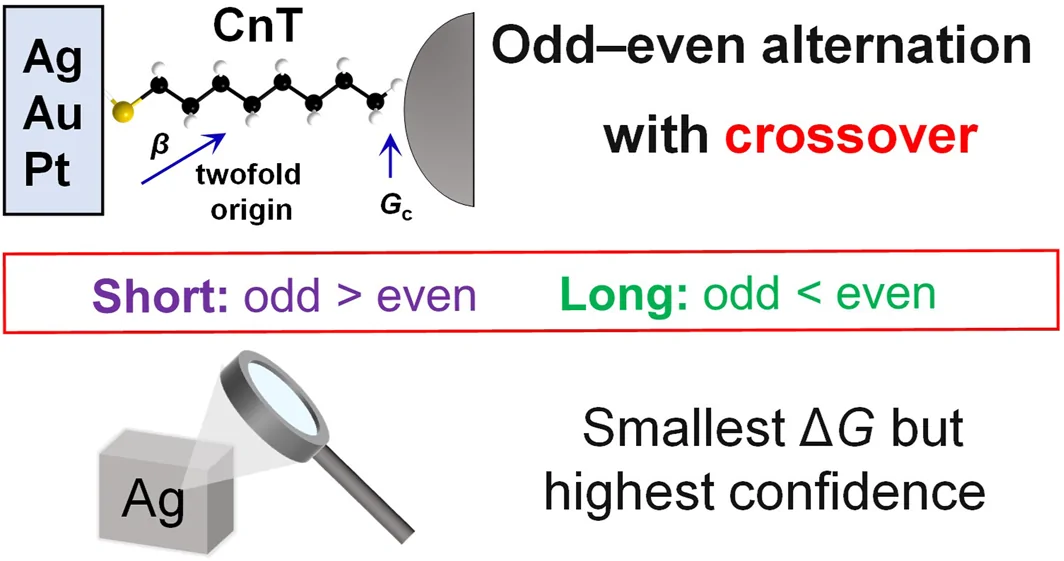

## 1 INTRODUCTION

First recognized nearly a century ago as a manifestation of quantum mechanical nature at the molecular scale,[^1^, ^2^, ^3^, ^4^] odd‐even alternation of various properties upon stepwise increasing the number of constituent particles has been reported for broad classes of systems, including multilayers,^[5]^ self‐assembled monolayers (SAMs),^[6]^ quantum dot arrays,^[7]^ and carbon chains.[^8^, ^9^, ^10^, ^11^]

Closer to the systems investigated in this paper, odd‐even effects were also observed in charge transport through large‐area molecular junctions[^12^, ^13^, ^14^, ^15^, ^16^, ^17^] fabricated with SAMs^[18]^ of alkanethiols (CnT)[^19^, ^20^] with variable number (n) of methylene units, where the thiol end anchors on metal and a top EGaIn contact contacts the methyl terminus.[^21^, ^22^, ^23^] Various explanations for the origin of the odd‐even effect in transport have been proposed. Some authors speculated on the role of interfaces via different conformational disorders near the chain termini or different preferential orientation of the terminal group,^[6]^ resulting in diverse binding to the top electrode[^21^, ^22^, ^23^] and possible differences in tunneling barriers[^23^, ^24^, ^25^, ^26^, ^27^, ^28^] and surface dipoles due to diverse chemical environments of the ${\text{CH}}_{3}$ groups in odd versus even SAMs.^[20]^ Inclined to assume a bulk rather than interface origin, other authors considered the influence of van der Waals forces between molecules,[^21^, ^22^, ^29^, ^30^] the geometry of the n‐alkyl chain, and the number of gauche bond conformers associated with the deficiency of electron transport.[^21^, ^22^, ^23^, ^31^]

By and large, the odd‐even effect remains a challenge in molecular electronics, with four key open questions:

(i) Platform dependence: Is the odd‐even alternation in CnT junctions platform‐dependent? It has been observed for large‐area EGaIn‐based junctions[^23^, ^25^, ^32^, ^33^, ^34^, ^35^, ^36^, ^37^, ^38^] but, to date, not reported in junctions fabricated with the conducting probe atomic force microscope (conducting probe atomic force microscopy (CP‐AFM)) platform.
(ii) Conductance trend: Are odd junctions more conductive than even junctions (as claimed in fig. 5 of Ref. [23] and fig. 2 of Ref. [35]) or vice versa (as claimed in fig. 4 of Ref. [34])?
(iii) Metal specificity: Is the odd‐even effect absent or at least weaker^[36]^ for silver electrodes than for gold electrodes?
(iv) Physical origin: Is this effect due to the interface or rather to the bulk?

Using extensive CP‐AFM measurements on CnT SAMs (n=7–12) with Ag, Au, and Pt electrodes (Figure 1), together with statistically rigorous analysis of parameters extracted from the off‐resonant single‐level model (see Supporting Information S1: Figures S1 and S2 and discussion related to it),^[39]^ the present study offers a Solomonic resolution to these issues. We show that the truth lies on both sides of each apparent dichotomy: the conductance superiority alternates with chain length, and the effect arises from both interface and bulk.[^6^, ^19^, ^20^, ^21^, ^22^, ^23^, ^29^, ^30^, ^31^]

**FIGURE 1:**
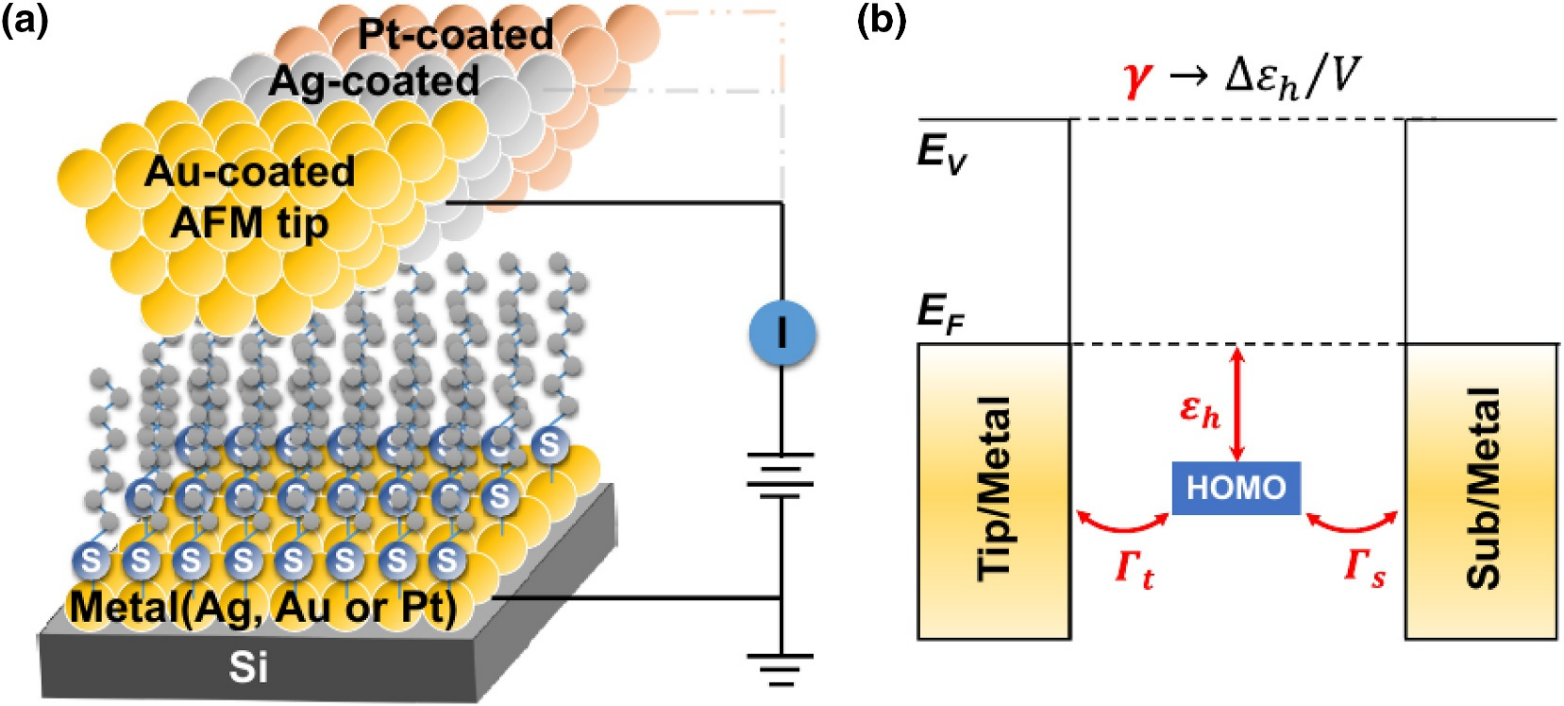
(a) Schematic representation of the CP‐AFM setup. A metal‐coated (Ag, Au, Pt) AFM tip approaches a SAM of CnT (n=7–12) adsorbed on a substrate coated with Ag, Au, or Pt. (b) Characteristic junction electronic structure: HOMO energy offset $\varepsilon_{h}$, molecular orbital electronic couplings $\Gamma_{s,t}$ to tip and substrate, and Stark effect strength γ. CP‐AFM, conducting probe atomic force microscopy; HOMO, highest occupied molecular orbital; SAM, self‐assembled monolayer.

## 2 RESULTS AND DISCUSSION

### 2.1 Conductance analysis

The conductances G of the CnT junctions underlying the present analysis were calculated by averaging the low‐bias slope of approximately 100–150 I–V traces measured similarly to our previous reports for other SAM systems.[^40^, ^41^, ^42^, ^43^, ^44^] Using these G values (Table 1) and highest occupied molecular orbital (HOMO) energy offsets relative to the metal's Fermi level $\varepsilon_{h}=E_{F}-E_{\text{HOMO}}>0$ determined by combining transition voltage spectroscopy with the off‐resonant single‐level model,[^39^, ^45^, ^46^] we determined the effective electronic MO‐electrode coupling Γ (details in Supporting Information S1). The number of molecules per junction N=80 needed to estimate Γ was deduced via contact mechanics^[47]^ combined with Rutherford backscattering and nuclear reaction analysis^[48]^ as elaborated earlier.^[40]^ These results, which constitute the input information for the analysis that follows, are collected in Table 1. For context, the extracted conductances G and effective couplings Γ are consistent with our recent extractions for CnT CP‐AFM junctions on Ag, Au, and Pt electrodes (see tab. S1 in Ref. [49]).

**TABLE 1:** Key electronic structure parameters.

Metal	Quantity	C7T	C8T	C9T	C10T	C11T	C12T
Ag	G (S)	$1.36\times 10^{-8}$	$2.38\times 10^{-9}$	$1.02\times 10^{-9}$	$2.59\times 10^{-10}$	$9.86\times 10^{-11}$	$2.74\times 10^{-11}$
	$\varepsilon_{h}$ (eV)	1.19	1.13	1.14	1.13	1.14	1.14
	Γ (meV)	1.76	0.70	0.46	0.23	0.14	0.08
Au	G (S)	$1.62\times 10^{-7}$	$3.18\times 10^{-8}$	$1.18\times 10^{-8}$	$3.26\times 10^{-9}$	$1.34\times 10^{-9}$	$3.51\times 10^{-10}$
	$\varepsilon_{h}$ (eV)	1.01	1.01	0.94	0.97	0.98	0.98
	Γ (meV)	5.16	2.29	1.30	0.70	0.46	0.23
Pt	G (S)	$1.23\times 10^{-6}$	$2.18\times 10^{-7}$	$8.55\times 10^{-8}$	$2.49\times 10^{-8}$	$9.19\times 10^{-9}$	$2.44\times 10^{-9}$
	$\varepsilon_{h}$ (eV)	0.91	0.91	0.88	0.83	0.88	0.87
	Γ (meV)	12.82	5.40	3.27	1.66	1.07	0.55

*Note*: Energy offset $\varepsilon_{h}$, junction conductance G, and effective molecule‐electrode coupling Γ for CnT self‐assembled monolayers on Ag, Au, and Pt computed from measured I–V curves using the single‐level model.

Supporting Information S1: Figure S3A, Table S1, and Equation (1) reflect the present knowledge on the transport properties of CP‐AFM junctions with Ag, Au, and Pt.[^42^, ^50^, ^51^] Data of conductance G and MO‐electrode coupling Γ exhibit an overall exponential decay with molecular size n, a signature of charge transport by off‐resonant tunneling.[^52^, ^53^, ^54^]

(1) $$G=G_{c}\;\exp\left(-\beta n\right),\ln G=\ln G_{c}\;-\;\beta n$$

This dependence holds for each metal investigated with metal‐specific prefactors $G_{c}$ (Supporting Information S1: Table S1).

The effect of the metal can be accounted for via the work function Φ, on which the contact conductance exponentially depends $G_{c}={\bar{G}}_{c}\;\exp\left(\xi \Phi \right)$ ($\Phi_{\text{Ag}}=4.25$ eV, $\Phi_{\text{Au}}=5.20$ eV, $\Phi_{\text{Pt}}=5.65$ eV).[^51^, ^55^, ^56^, ^57^, ^58^, ^59^]

(2) $$G={\bar{G}}_{c}\;\exp\left(-\beta n+\xi \Phi \right),\ln G=\ln{\bar{G}}_{c}\;-\;\beta n+\xi \Phi$$

Above, the parameter ξ quantifies the conductance's work function sensitivity. Data fitting to Equation (2) are presented in Supporting Information S1: Table S1 and Figure S4A.

Supporting Information S1: Figures S3 and S4 along with the numerical values in Supporting Information S1: Table S1 emerging from Equations (1) and (2) capture the overall exponential decay but necessarily average over any parity‐dependent structure; the resulting $R^{2}$ values, while still high (≈0.99), are systematically lower than those obtained when odd and even series are treated separately. This possibility is addressed by parity‐splitting of Equation (1), that is, allowing odd and even members to have different properties (p=o,e)

(3) $$G_{p}=G_{c,p}\;\exp(-\beta_{p}n),\ln G_{p}=\ln G_{c,p}\;-\;\beta_{p}n$$

with model parameters ($G_{c,p}$, $\beta_{p}$) adjusted to individual metals. This parity‐splitting fitting approach yielded the results collected in Table 2 and depicted in Figure 2a. The improvement in fit quality (especially the near‐perfect linearity of the even series) and the statistically significant separation of the parity‐specific parameters demonstrate that analyzing the odd and even datasets separately provides a demonstrably better description of the data than a single, parity‐agnostic model.

**TABLE 2:** Parity‐dependent fitting results and statistical significance for conductance G using Equation (3) $\left(G_{p}=G_{c,p}\;\exp(-\beta_{p}n)\right)$.

Metal	Parity	$\ln G_{c,p}$	$\beta_{p}$	$R^{2}$
Ag	Odd	−9.53 ± 0.34	1.232 ± 0.037	0.9991
Ag	Even	−10.92 ± 0.04	1.116 ± 0.004	0.99999
Au	Odd	−7.32 ± 0.59	1.199 ± 0.064	0.9971
Au	Even	−8.26 ± 0.07	1.127 ± 0.007	0.99996
Pt	Odd	−5.11 ± 0.58	1.224 ± 0.063	0.9974
Pt	Even	−6.33 ± 0.22	1.123 ± 0.022	0.9996

Metal	Conf. $\ln G_{c,o}>\ln G_{c,e}$	z‐score $\left(\ln G_{c}\right)$	p‐value $\left(\ln G_{c}\right)$	Conf. $\beta_{o}>\beta_{e}$	z‐score (β)	p‐value (β)
Ag	99.999	+4.11	$1.94\times 10^{-5}$	99.91	+3.14	$8.54\times 10^{-4}$
Au	94.4	+1.59	0.056	86.8	+1.12	0.132
Pt	97.6	+1.97	0.024	93.5	+1.52	0.065

*Note*: Top panel: metal‐ and parity‐dependent best‐fit parameters ($\ln G_{c,p}$, $\beta_{p}$, $R^{2}$). Bottom panel: confidence levels (%), z‐scores and p‐values testing whether odd chains have larger G‐related properties than even chains.

**FIGURE 2:**
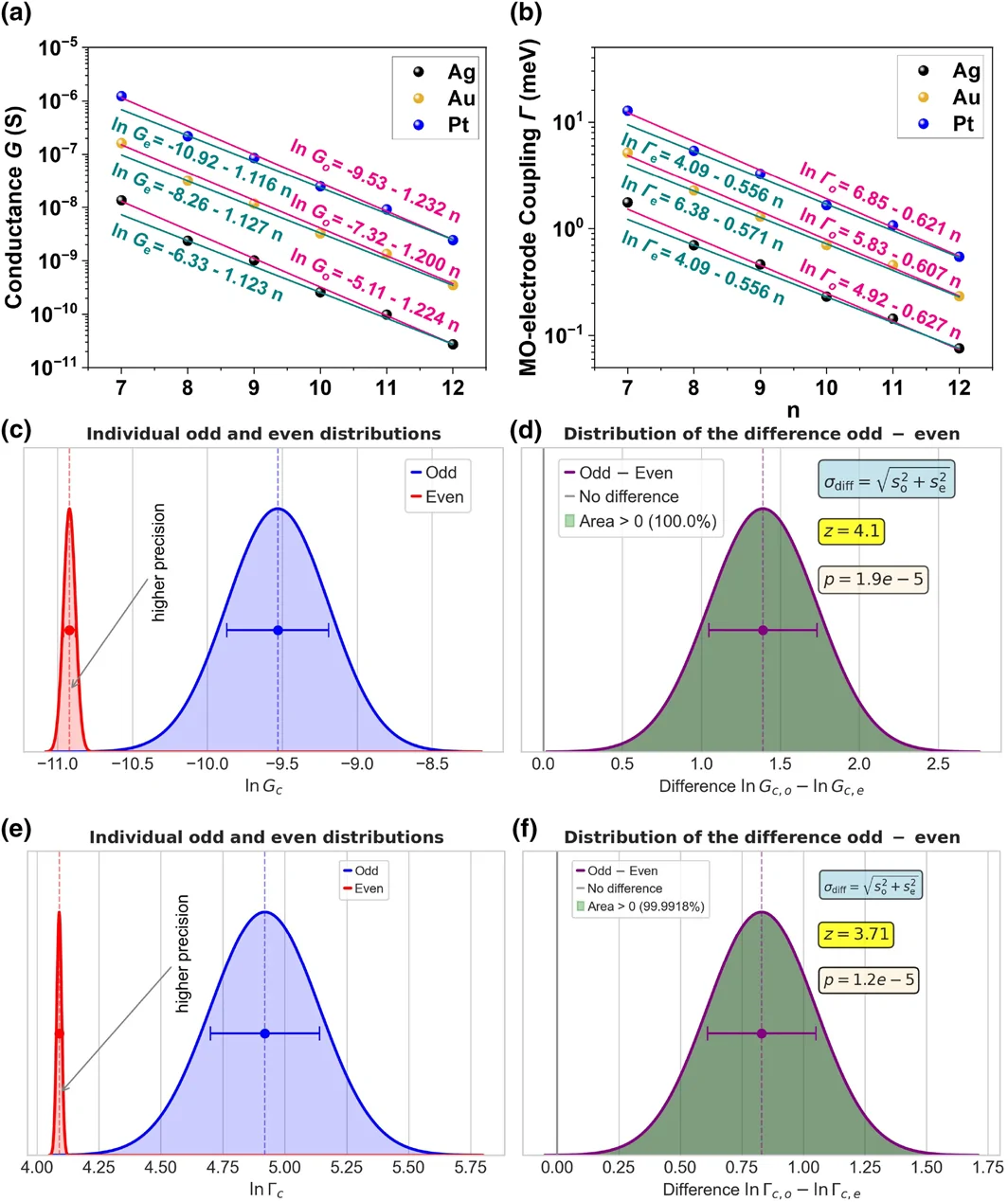
Parity‐separated analysis of transport data for CnT junctions with Ag, Au, and Pt electrodes. (Left) Conductance G fitted to Equation (3); (Right) Highest occupied molecular orbital‐electrode coupling Γ fitted to Supporting Information S1: Equation (S14). Upper panels: Semilog plots of experimental data with best‐fit lines for odd and even junctions. Best‐fit parameters—prefactors ($G_{c,p}$, $\Gamma_{c,p}$) and length attenuation coefficients ($\beta_{p}$, ${\bar{\beta }}_{p}$)—are metal‐ and parity‐dependent; numerical values are given in Tables 2 and 4 and legends. Middle panels: Individual distributions of odd and even members for Ag junctions, showing the separation between parities. Bottom panels: Distributions of the difference (odd − even) for Ag junctions. The z‐scores and one‐sided p‐values (from Tables 2 and 4) are indicated in the legends. The green shaded area in the bottom panels represents the probability that odd > even; its near‐complete coverage of the distribution demonstrates overwhelming statistical evidence for the odd‐even effect in Ag junctions. All parameter distributions are constructed as normal distributions centered on the least‐squares estimates with widths equal to the reported standard errors; they are therefore parametric illustrations of the uncertainty that enters the z‐score calculation rather than nonparametric bootstrap resamples of the raw I–V traces.

A quick glance at these results may not suffice to conclusively establish the existence of an odd‐even alternation effect. Although “reasonably” parity‐dependent, the differences between the best‐fit parameters might not prevail over the relatively large standard deviations.

A closer look at Table 2 reveals that standard errors for even‐parity fits are substantially smaller than those for odd‐parity fits (e.g., ±0.04 vs. ±0.34 for $\ln G_{c}$ on Ag). This is an important observation. The aforementioned disparity necessitates a statistical approach that accounts for unequal variances to fairly compare the two groups, a feature that cannot be adequately accounted for by the common Student's t‐test.

### 2.2 Statistical assessment of parity (odd‐even) differences

Therefore, to assess whether the differences between odd and even parameters are statistically significant, we employ the z‐score method, equivalent to Welch's t‐test for unequal variances. All necessary details are presented in Supporting Information S1.

This approach is crucial because most experimentalists rely solely on best‐fit parameters and their standard deviations, which does not rigorously determine whether two quantities are truly different. The z‐score is expressed in terms of the best fitting values (e.g., $\ln G_{c,o}$ and $\ln G_{c,e}$) and their standard errors. The Welch p‐value is derived from the standard normal distribution. We primarily report one‐sided confidence levels because the directional hypothesis (odd > even for both prefactor and β) is physically motivated by the consistent trend observed across all three metals and by the expected structural consequences of parity (terminal‐group orientation and packing). To address possible concerns of circular reasoning, we also evaluated the more conservative two‐sided tests. For Ag the two‐sided p‐values remain extremely small ($p_{\text{two}‐\text{sided}}=3.9\times 10^{-5}$ for $\ln G_{c}$ and $1.7\times 10^{-3}$ for β), corresponding to confidence levels >99.99% and >99.8%, respectively. Thus the central claim of a statistically robust odd–even effect on Ag is unaffected by the choice of one‐ versus two‐sided testing. High confidence (>95%) indicates a real difference. (Recall that ubiquitous comparison of data with standard (1 − σ) error bars amounts to 68% confidence.)

The results of the analysis based on Equation (3) collected in the lower part of Table 2 demonstrate:

- For Ag, the evidence is overwhelming: the prefactor difference exceeds four times the combined uncertainty (z=4.11, one‐sided p<0.00002; two‐sided $p=3.9\times 10^{-5}$), and the decay constant difference is also highly significant (z=3.14, one‐sided p<0.001; two‐sided $p=1.7\times 10^{-3}$).
- For Pt, the evidence is strong for the prefactor (97.6% one‐sided confidence) and suggestive for the decay constant (93.5%).
- For Au, the prefactor difference approaches conventional significance (94.4%), while the decay constant difference is weaker but still trends in the same direction.

The even‐parity chains exhibit exceptionally clean exponential behavior across all three metals, with $R^{2}$ values very close to 1.000 and standard errors typically below 0.04 (in units of lnG), indicating highly reproducible measurements with low scatter. In contrast, the odd‐parity fits show slightly larger residuals, yet still maintain excellent linearity $\left(R^{2}\geq 0.997\right)$.

Across all metals, odd‐parity chains are characterized by a larger (less negative) prefactor $\ln G_{c}$ and a larger attenuation coefficient β compared to even‐parity chains. This pattern implies that odd chains tend to exhibit higher conductance at short lengths but decay more rapidly with increasing n. The separation is most pronounced for Ag, where $\ln G_{c,o}=-9.53\pm 0.34$ versus $\ln G_{c,e}=-10.92\pm 0.04$, and $\beta_{o}=1.23\pm 0.04$ versus $\beta_{e}=1.12\pm 0.004$.

### 2.3 Incorporating work function dependence

We now investigate whether the metal dependence can be accounted for via an exponential term in Φ, as observed in conjunction with Equation (2). To this aim, we analyzed the data using a hierarchy of models with progressively fewer free parameters.

*Global parity‐split model with metal‐dependent decay*: First, a more constrained model is fitted across all metals simultaneously while preserving parity‐specific and metal‐specific features (p=o,e)

(4) $$G_{p}={\bar{G}}_{c,p}\;\exp\left(-\beta_{p,m}n+\xi_{p}\Phi \right),\;\ln G_{p}=\ln{\bar{G}}_{c,p}-\beta_{p,m}n+\xi_{p}\Phi$$

where ${\bar{G}}_{c,p}$ and $\xi_{p}$ are parameters shared by all metals for each parity p (odd or even), $\beta_{p,m}$ varies independently for each metal m and parity p, and Φ is the electrode work function. This formulation explicitly accounts for work‐function‐dependent attenuation factor (β) while testing whether a single parity‐dependent prefactor and work function sensitivity (ξ) can describe the entire dataset.

The global prefactor remains larger (less negative) for odd chains, although the statistical separation is weaker than in the per‐metal fits of Equation (3). The work‐function sensitivity parameters $\xi_{o}$ and $\xi_{e}$ show no meaningful difference, indicating that the influence of electrode work function on the effective energy offset is essentially the same for odd‐ and even‐parity chains; the negative value of confidence z=−0.31 corresponds to a confidence of 38% (green shaded area) while confidence that $\xi_{e}>\xi_{o}$ is 62% (pink shaded area in the right bottom panel of Figure 3)—essentially indistinguishable from chance. In contrast, the metal‐ and parity‐dependent attenuation coefficients retain a clear pattern: $\beta_{o}>\beta_{e}$ for every metal, with strong statistical support on Ag and Au (middle panels in Figure 3), and a suggestive trend on Pt.

**FIGURE 3:**
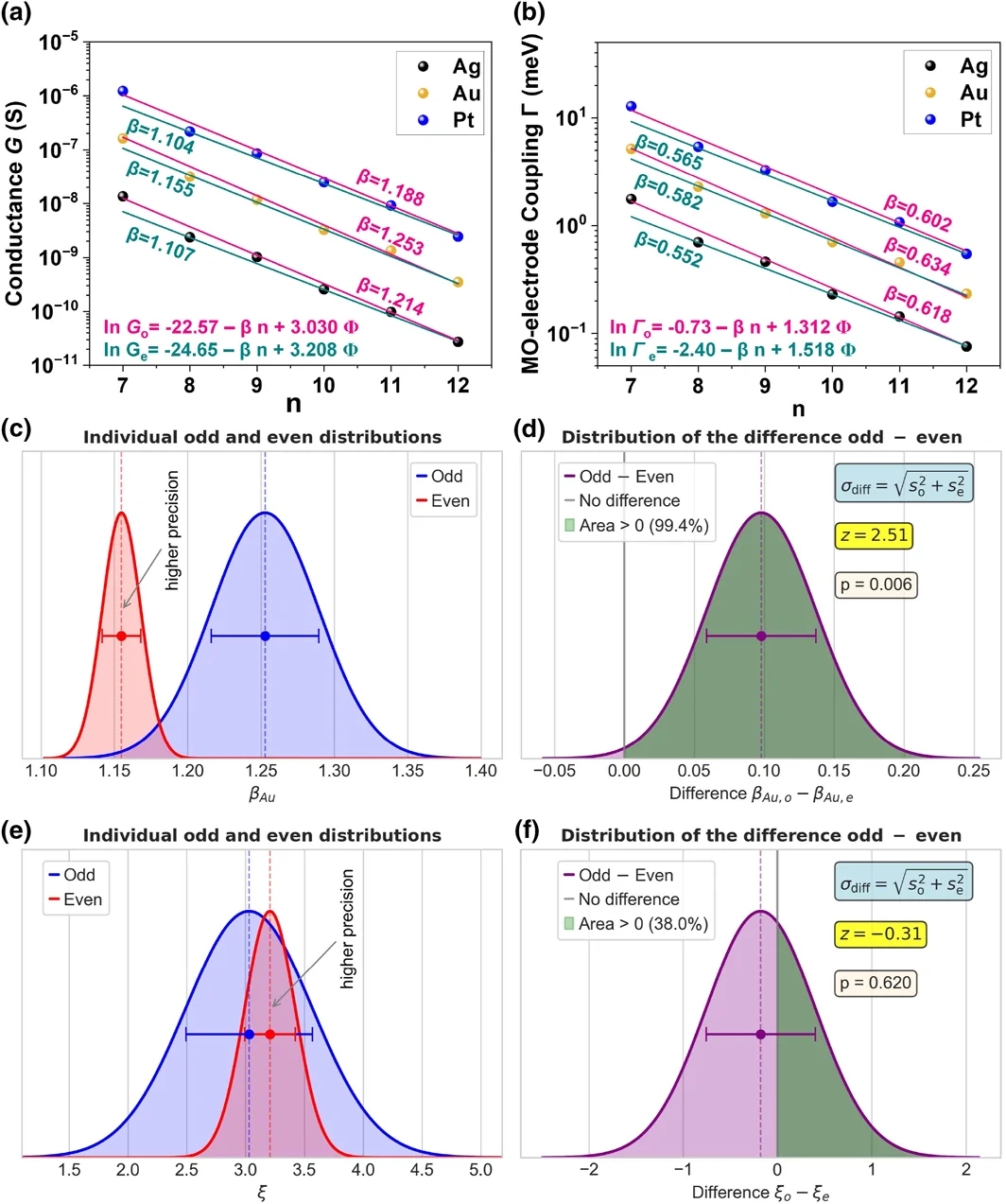
Parity‐separated global fits incorporating work‐function dependence with metal‐dependent decay coefficients. Upper panels: (a) Conductance G fitted to Equation (4) and (b) highest occupied molecular orbital‐electrode coupling Γ fitted to Supporting Information S1: Equation (S15) for Ag, Au, and Pt electrodes. Prefactors (${\bar{G}}_{c,p}$, ${\bar{\Gamma }}_{c,p}$) and work‐function sensitivities ($\xi_{p}$, ${\bar{\xi }}_{p}$) are parity‐dependent but metal‐independent, while decay coefficients ($\beta_{p,m}$, ${\bar{\beta }}_{p,m}$) vary with both parity and metal. Middle panels: Distributions of odd and even decay coefficients for Au junctions (left) and the corresponding difference distribution (right). The pink shaded area (0.6% of the distribution) represents the probability that $\beta_{\text{Au},o}<\;\beta_{\text{Au},e}$; conversely, $\beta_{\text{Au},o}>\beta_{\text{Au},e}$ holds with 99.4% confidence (green shaded area, cf. Table 3). Bottom panels: Distributions of odd and even work‐function sensitivities (left) and their difference (right). Unlike the decay coefficients (β), $\xi_{o}>\xi_{e}$ holds with only 38% confidence (negative z‐score, green shaded area), indicating that the apparent reversal $\left(\xi_{e}>\xi_{o}\right)$ is statistically indistinguishable from chance. This confirms that work‐function sensitivity is parity‐insensitive within experimental precision. Numerical values are given in Tables 3 and 5.

**TABLE 3:** Parity‐dependent fitting results and statistical significance for conductance G using Equation (4) $\left(G_{p}={\bar{G}}_{c,p}\;\exp(-\beta_{p,m}n+\xi_{p}\Phi )\right)$.

Parity	$\ln{\bar{G}}_{c}$	ξ (${\text{eV}}^{-1}$)	$\beta_{\text{Ag}}$	$\beta_{\text{Au}}$	$\beta_{\text{Pt}}$	$R^{2}$
Odd	−22.57 ± 2.72	+3.030 ± 0.538	1.214 ± 0.057	1.253 ± 0.037	1.188 ± 0.050	0.9983
Even	−24.65 ± 1.09	+3.208 ± 0.215	1.107 ± 0.021	1.155 ± 0.013	1.104 ± 0.018	0.9998

Parameter	Confidence (%)	z‐score	p‐value
$\ln{\bar{G}}_{o}>\ln{\bar{G}}_{e}$	76.0	+0.71	0.240
$\xi_{o}>\xi_{e}$	38.0	−0.31	0.620
$\beta_{o}>\beta_{e}$ (Ag)	96.0	+1.76	0.040
$\beta_{o}>\beta_{e}$ (Au)	99.4	+2.51	0.006
$\beta_{o}>\beta_{e}$ (Pt)	94.1	+1.56	0.059

*Note*: Top panel: metal‐ and parity‐dependent best‐fit parameters ($\ln G_{c,p}$, $\beta_{p}$, $R^{2}$). Bottom panel: confidence levels (%), z‐scores and p‐values testing whether odd chains have larger G‐related properties than even chains.

*Full parity‐split model*: In the next step, we perform completely independent regressions for the odd and even datasets across all metals and chain lengths

(5) $$G_{p}={\bar{G}}_{c,p}\;\exp\left(-\beta_{p}n+\xi_{p}\Phi \right),\;\ln G_{p}=\ln{\bar{G}}_{c,p}-\beta_{p}n+\xi_{p}\Phi$$

where ${\bar{G}}_{c,p}$, $\beta_{p}$, and $\xi_{p}$ are fitted separately for each parity, without any parameter sharing.

The global prefactor is again larger for odd chains, with moderate statistical support. The attenuation coefficient follows the same trend $\left(\beta_{o}>\beta_{e}\right)$, while the work‐function coefficients are essentially indistinguishable. This null result for $\Delta \xi =\xi_{o}-\xi_{e}$ reinforces that parity‐dependent differences in electrode work‐function coupling are not detectable within the precision of the present measurements.

*Parity‐split model with global work‐function sensitivity*: This model imposes the strongest physical constraint by sharing a single global coefficient ξ across both parities while allowing parity‐specific prefactors and decay constants

(6) $$G_{p}={\bar{G}}_{p}\;\exp\left(-\beta_{p}n+\xi \Phi \right),\;\ln G_{p}=\ln{\bar{G}}_{p}\;-\;\beta_{p}n+\xi \Phi$$

This formulation assumes that the sensitivity of the effective energy offset to electrode work function is parity‐independent—a reasonable hypothesis if parity differences arise primarily from molecular conformation or contact geometry rather than from systematic differences in level alignment (more details in section “Mechanistic Interpretation” of Supporting Information S1).

The parity‐specific parameters follow the pattern observed in earlier steps: a larger prefactor and steeper decay for odd chains.

The progression across the various steps demonstrates robust convergence: regardless of how work‐function dependence is modeled, the core parity signature persists—a larger extrapolated prefactor for odd chains combined with faster length‐dependence decay.

### 2.4 Odd‐even crossover

Inspection of Figures 2, 3, 4 reveals that, regardless of the model, odd chains exhibit both larger intercepts $\left(\ln G_{c,o}>\ln G_{c,e}\right)$ and larger slopes $\left(\beta_{o}>\beta_{e}\right)$. This necessarily implies the existence of a crossover length $n_{\text{cross}}$ such that:

(7) $$\left\{\begin{array}{cc} G_{o}>G_{e} & \;\text{for}\;n<n_{\text{cross}}\\ G_{o}<G_{e} & \;\text{for}\;n>n_{\text{cross}} \end{array}\right.$$

**FIGURE 4:**
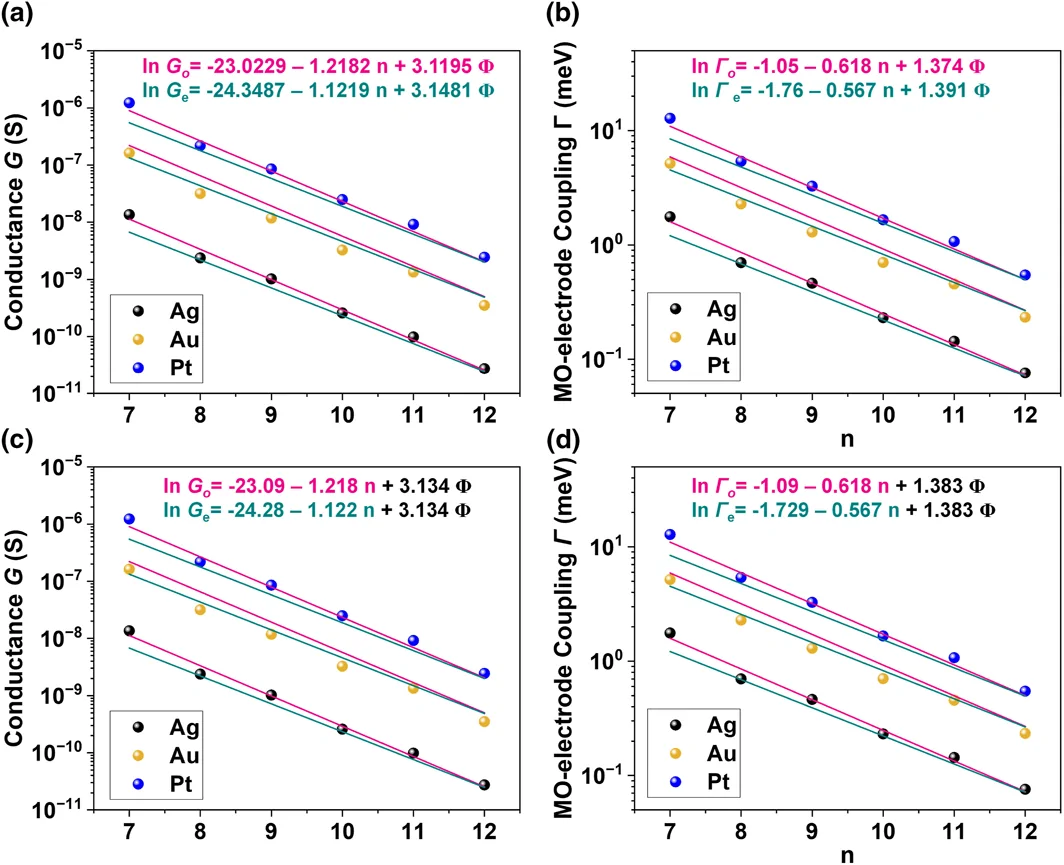
Parity‐separated global fits with metal‐independent decay coefficients. Upper half: Full parity‐split model (Equation 5 and Supporting Information S1: Equation S16), where prefactors (${\bar{G}}_{c,p}$, ${\bar{\Gamma }}_{c,p}$), decay coefficients ($\beta_{p}$, ${\bar{\beta }}_{p}$), and work‐function sensitivities ($\xi_{p}$, ${\bar{\xi }}_{p}$) are all parity‐dependent but metal‐independent. Lower half: Constrained model (Equation 6 and Supporting Information S1: Equation S17) sharing a single global work‐function sensitivity ξ (or $\bar{\xi }$) across both parities while retaining parity‐dependent prefactors and decay coefficients. (a) and (c) Conductance G; (b) and (d) highest occupied molecular orbital‐electrode coupling Γ. Across both formulations, the core parity signature persists: odd chains exhibit larger prefactors (higher short‐chain conductance) but steeper decay coefficients, while work‐function sensitivity shows no meaningful parity dependence. Numerical values are given in Supporting Information S1: Tables S2, S3, S5 and S6.

The existence of such a crossover is a robust qualitative finding of this work: the relative conductance of odd versus even chains inverts with increasing molecular length. This single observation reconciles seemingly contradictory literature reports,[^34^, ^35^] which likely correspond to measurements on opposite sides of this crossover.

Quantitatively, because the work‐function sensitivity ξ is parity‐independent within statistical uncertainty, the crossover condition $G_{o}=G_{e}$ in Equation (6) yields:

(8) $$n_{\text{cross}}=\frac{\ln{\bar{G}}_{c,e}\;-\;\ln{\bar{G}}_{c,o}}{\beta_{o}\;-\;\beta_{e}}$$

Using the parameters from our global fit (Supporting Information S1: Table S2), we obtain a point estimate of $n_{\text{cross}}\approx 12.3$. However, this value should be interpreted with considerable caution. The denominator $\beta_{o}-\beta_{e}$ is small (≈0.1), making the estimate highly sensitive to minor uncertainties in the fitted slopes. Moreover, our experimental window (n=7–12) lies entirely around the estimated crossover, precluding precise extrapolation. A Monte Carlo uncertainty propagation (detailed in Supporting Information S1 with accompanying Python code) yields a 95% confidence interval of approximately [5.4, 33.6], confirming that while the existence of a crossover is robust, its precise value cannot be reliably determined from the present data.

What is important—and statistically robust—is the qualitative conclusion: odd chains dominate at short lengths, even chains at longer lengths. The exact crossover length may depend on specific junction details (metal work function, contact geometry, measurement conditions) and could vary between experimental platforms. Future studies with longer chains (n>12) would be valuable to locate the crossover more precisely.

### 2.5 Parity dependence in molecule–electrode electronic coupling Γ

The off‐resonant single‐level model provides an adequate framework for extracting electronic parameters from measured transport characteristics (details in Supporting Information S1).^[39]^ Within this model, the low‐bias conductance $G_{n}$ of a CnT junction containing N molecules is expressed in terms of the HOMO energy offset $\varepsilon_{h,n}=E_{F}-E_{\text{HOMO},n}$ and the effective HOMO‐electrode coupling $\Gamma_{n}$:

The effective molecule–electrode electronic coupling $\Gamma =\sqrt{\Gamma_{s}\Gamma_{t}}$ (where $\Gamma_{s,t}$ are the HOMO couplings to the substrate and tip, respectively) was extracted from the measured I–V characteristics using the off‐resonant single‐level model^[39]^ combined with transition voltage spectroscopy[^45^, ^46^] (see Supporting Information S1: Equation (S4) and further details in Supporting Information S1).

Inspection of Table 1 reveals that the HOMO offset $\varepsilon_{h}$ remains essentially independent of n for each metal (maximum variation 0.07 eV across n=7–12). Consequently, the observed exponential decay of conductance G with molecular length n must stem predominantly from the exponential weakening of the HOMO‐tip electronic coupling $\Gamma_{t,n}$

$$\tau_{t,n}\;\approx \;\tau_{t,c}\;e^{-\beta n/2}\;\Leftrightarrow \;\Gamma_{t,n}\;\approx \;\Gamma_{t,c}\;e^{-\beta n}\;\Leftrightarrow \;\Gamma_{n}\;\approx \;\Gamma_{c}\;e^{-\beta n/2}.$$

It follows that lnG∝−βn and $\ln\Gamma \;\propto -\bar{\beta }n$ with $\bar{\beta }\;\approx \;\beta /2$, a relation directly supported by the subsequent fits.

With this understanding, we now analyze the parity effect on the MO‐electrode coupling Γ. The same model hierarchy applied to lnG was used for lnΓ: Supporting Information S1: Equations (S12–S17), with relevant results in Tables 4 and 5, Supporting Information S1: Tables S5 and S6, and Figures 2, 3, 4.

**TABLE 4:** Parity‐dependent fitting results and statistical significance for Γ using Supporting Information S1: Equation (S14) $\left(\Gamma_{p}=\Gamma_{c,p}\;\exp\left(-{\bar{\beta }}_{p}n\right)\right)$.

Metal	Parity	$\ln{\bar{\Gamma }}_{c,p}$	${\bar{\beta }}_{p}$	$R^{2}$
Ag	Odd	+4.92 ± 0.22	0.627 ± 0.025	0.9985
Ag	Even	+4.09 ± 0.01	0.556 ± 0.001	0.999998
Au	Odd	+5.83 ± 0.44	0.607 ± 0.048	0.9937
Au	Even	+5.38 ± 0.11	0.571 ± 0.011	0.9996
Pt	Odd	+6.85 ± 0.33	0.620 ± 0.036	0.9966
Pt	Even	+6.26 ± 0.09	0.573 ± 0.009	0.9998

Metal	Conf. $\ln\Gamma_{c,o}>\ln\Gamma_{c,e}$ (%)	z‐score (A)	p‐value (A)	Conf. ${\bar{\beta }}_{o}>{\bar{\beta }}_{e}$ (%)	z‐score $\left(\bar{\beta }\right)$	p‐value $\left(\bar{\beta }\right)$
Ag	99.99	+3.71	$1.02\times 10^{-4}$	99.80	+2.88	$1.96\times 10^{-3}$
Au	83.9	+0.99	0.161	76.6	+0.73	0.234
Pt	95.8	+1.73	0.042	89.9	+1.27	0.101

*Note*: Top panel: metal‐ and parity‐dependent best‐fit parameters ($\ln\Gamma_{c,p}$, $\beta_{p}$, $R^{2}$). Bottom panel: confidence levels (%), z‐scores and p‐values testing whether odd chains have larger Γ‐related properties than even chains.

**TABLE 5:** Parity‐dependent fitting results and statistical significance for MO‐electrode coupling Γ using Supporting Information S1: Equation (S15) $\left(\Gamma_{p}={\bar{\Gamma }}_{c,p}\;\exp\left(-{\bar{\beta }}_{p,m}n+{\bar{\xi }}_{p}\Phi \right)\right)$.

Parity	$\ln{\bar{\Gamma }}_{c,p}$	$\bar{\xi }$ (${\text{eV}}^{-1}$)	${\bar{\beta }}_{p,\text{Ag}}$	${\bar{\beta }}_{p,\text{Au}}$	${\bar{\beta }}_{p,\text{Pt}}$	$R^{2}$
Odd	−0.73 ± 1.70	+1.312 ± 0.335	0.618 ± 0.036	0.634 ± 0.023	0.602 ± 0.031	0.9971
Even	−2.40 ± 0.51	+1.518 ± 0.100	0.552 ± 0.010	0.582 ± 0.006	0.565 ± 0.008	0.9998

Parameter	Confidence (%)	z‐score	p‐value
$\ln{\bar{\Gamma }}_{c,o}>\ln{\bar{\Gamma }}_{c,e}$	82.6	+0.94	0.174
${\bar{\xi }}_{o}>{\bar{\xi }}_{e}$	27.7	−0.59	0.723
${\bar{\beta }}_{o}>{\bar{\beta }}_{e}$ (Ag)	96.1	+1.77	0.039
${\bar{\beta }}_{o}>{\bar{\beta }}_{e}$ (Au)	98.6	+2.21	0.014
${\bar{\beta }}_{o}>{\bar{\beta }}_{e}$ (Pt)	87.1	+1.13	0.129

*Note*: Top panel: best‐fit parameters ($\ln{\bar{\Gamma }}_{c,p}$, ${\bar{\xi }}_{p}$, metal‐dependent ${\bar{\beta }}_{p,m}$, $R^{2}$). Bottom panel: confidence levels (%), z‐scores and p‐values testing whether odd chains have larger Γ‐related properties than even chains.

Across all formulations, fits are of high quality Supporting Information S1: Figures S3 and S4, and Figures 2, 3, 4, with even‐parity chains again showing particularly low scatter.

In the least constrained per‐metal/per‐parity fits (Supporting Information S1: Equation S14), the prefactor $\ln\Gamma_{c}$ is systematically larger for odd chains on all metals, with very strong evidence on Ag and solid support on Pt. The attenuation coefficient $\bar{\beta }$ is also larger for odd chains (faster decay of coupling with length), most convincingly on Ag. These trends mirror the behavior observed for G, albeit with somewhat weaker statistical separation on Au and Pt.

Progressive model constraints (Supporting Information S1: Equations S15–S17) preserve the qualitative pattern: $\ln\Gamma_{c,o}>\ln\Gamma_{c,e}$ and ${\bar{\beta }}_{o}>{\bar{\beta }}_{e}$, while the work‐function sensitivity $\bar{\xi }$ shows no significant parity dependence. This confirms that parity does not meaningfully modulate the response of coupling strength to electrode work function.

The convergence across models for Γ closely parallels the results for G: odd chains exhibit stronger short‐chain coupling (higher prefactor) but faster decay of that coupling with molecular length (higher $\bar{\beta }$), while no robust parity effect appears in the work‐function dependence.

The consistent lack of significant parity dependence in the work‐function sensitivity (ξ, $\bar{\xi }$) strongly suggests that the observed odd‐even transport asymmetry is not primarily driven by differences in Fermi‐level alignment or electrode‐specific coupling strength. Instead, the results point toward structural origins—such as parity‐dependent average tilt angles, packing motifs, conformational disorder, or molecule‐electrode contact geometry—that simultaneously enhance short‐chain conductance/coupling (via larger prefactor $\left(G_{c},\Gamma_{c}\right)$) and increase the decay coefficient $\left(\beta ,\bar{\beta }\right)$ for odd chains.

The work‐function sensitivity $\xi \;\approx \;3.1\;{\text{eV}}^{-1}$ is parity‐independent, a finding that can be understood from the parity independence of the HOMO offset $\varepsilon_{h}$ (Table 1). A quantitative estimate from first principles is challenging because it requires convoluting the molecular HOMO wavefunction with the image potential, which is singular near the metal surface. Nevertheless, the HOMO spatial distribution is concentrated at the thiol end and is parity‐independent; any image‐driven shift therefore affects odd and even chains equally. The same argument applies to interface dipoles, whose magnitude is determined by the local chemistry at the thiol terminus. Thus, the parity independence of ξ supports a structural (rather than electrostatic) origin of the odd‐even effect. Further details are provided in section “Mechanistic Interpretation” of Supporting Information S1.

### 2.6 Answers to key questions

We now provide concise answers to the four questions posed in the Introduction:

(i) Platform dependence: Previously, the odd‐even effect was reported only for large area EGaIn‐based junctions. Under the conditions of the present CP‐AFM experiments the odd–even effect is clearly observed. Earlier CP‐AFM studies on CnT junctions[^42^, ^48^, ^53^, ^60^, ^61^, ^62^, ^63^] did not report the effect because the data were analyzed in a parity‐agnostic manner. When odd and even series are pooled, the high $R^{2}$ values of the overall exponential fit conceal the underlying alternation (Supporting Information S1: Tables S1 and S4; Figures S3 and S4); only deliberate parity separation reveals it. The experimental methods used here are essentially the same as those employed previously on polycrystalline electrodes,^[42]^ under which exceptionally small statistical variations in transport properties have already been demonstrated.^[40]^ The present observation of the odd–even effect therefore does not require invoking differences in electrode crystallinity (if anything, single‐crystal electrodes would enhance rather than suppress this effect) or other technical factors that would suppress the phenomenon; it arises from a more refined, parity‐resolved analysis of data of comparable quality.
(ii) Conductance trend: The relative conductance of odd versus even chains depends on chain length. Because $\ln G_{c,o}>\ln G_{c,e}$ but $\beta_{o}>\beta_{e}$, odd chains are more conductive at short lengths $\left(n<n_{\text{cross}}\right)$, while even chains become more conductive at longer lengths $\left(n>n_{\text{cross}}\right)$. This may reconcile the apparently contradictory reports in the literature[^34^, ^35^]—they may correspond to measurements on opposite sides of the crossover size $n_{c}$. The precise numerical value of $n_{\text{cross}}$ remains subject to substantial uncertainty owing to the limited chain‐length window.
(iii) Metal specificity: The odd‐even effect is present in all metals investigated (Ag, Au, Pt) but exhibits a striking and informative irony: although the effect is *weakest* in Ag in terms of absolute conductance differences, it is *strongest* in Ag in terms of statistical confidence.
  This apparent paradox is resolved by understanding the interplay between work function scaling and measurement precision. The exponential scaling G∝exp(ξΦ) with $\xi \;\approx \;3.1\;{\text{eV}}^{-1}$ means that conductances on Ag ($\Phi_{\text{Ag}}=4.25$ eV) are intrinsically lower than on Au ($\Phi_{\text{Au}}=5.2$ eV) by a factor of $e^{\xi \left(\Phi_{\text{Au}}-\Phi_{\text{Ag}}\right)}\;\approx \;e^{3.1\times 0.95}\approx \;19$. Consequently, the absolute odd‐even differences scale accordingly:
$$\frac{\left(G_{o}-G_{e}\right){|}_{\text{Au}}}{\left(G_{o}-G_{e}\right){|}_{\text{Ag}}}\;\approx \;e^{\xi \left(\Phi_{\text{Au}}-\Phi_{\text{Ag}}\right)}\;\approx \;19$$
  However, statistical confidence depends not on absolute differences but on the ratio of difference to uncertainty. The even‐parity Ag junctions exhibit exceptionally clean behavior, with standard errors an order of magnitude smaller than for odd‐parity Ag (e.g., $\sigma_{\ln G_{c,e}}=0.04$ vs. $\sigma_{\ln G_{c,o}}=0.34$). This exceptional reproducibility for even chains on Ag—combined with the consistent directional trend across all metals—yields z‐scores of 4.11 for the prefactor and 3.14 for β, corresponding to confidence levels exceeding 99.99% and 99.9%, respectively.
  Thus, the ironic outcome: the metal where the odd‐even effect is *smallest in magnitude* provides the *strongest statistical proof* of its existence. This demonstrates the power of proper statistical analysis—particularly Welch's t‐test for unequal variances—to extract robust conclusions even when absolute effects are small, provided measurement precision is high. It also underscores that the oft‐cited claim of weaker odd‐even effects for Ag^[36]^ reflects the smaller magnitude correctly but should not be misinterpreted as absence of the effect.
  A possible contribution of residual silver oxide (${\text{Ag}}_{x}$O) on the Ag electrodes cannot be entirely excluded under ambient conditions. However, several observations argue against oxide as the dominant source of the Ag‐specific statistical behavior. First and foremost, the even‐parity Ag series exhibits exceptionally low scatter $\left(R^{2}>0.99999\right)$, which would be difficult to reconcile with a disordered or chemically heterogeneous oxide layer. If significant, it would hurt rather than improve the statistical confidence. Second, the same directional parity trends (larger $G_{c}$ and β for odd chains) appear on Au and Pt, metals that do not form analogous surface oxides under the preparation and measurement conditions used here. Third, the work‐function scaling across Ag, Au, and Pt collapses onto a single exponential with $\xi \;\approx \;3.1\;{\text{eV}}^{-1}$, consistent with metallic rather than oxide‐dominated contacts. While a minor oxide contribution cannot be ruled out, it is unlikely to be the primary origin of the high statistical confidence observed for Ag.
(iv) Physical origin, a Solomonic answer: The effect arises from *both* interface and bulk contributions. The odd‐even alternation in the contact prefactors ($G_{c}$, $\Gamma_{c}$) is most naturally attributed to differences in molecule–electrode coupling at the termini (terminal‐group orientation, contact geometry), while the parity dependence of the decay constants (β, $\bar{\beta }$) points to differences in through‐chain propagation (average tilt, packing density, or conformational disorder). These assignments remain inferential, being drawn from the transport parameters themselves rather than from independent structural measurements on the same junctions. Direct microscopic evidence (e.g., NEXAFS tilt angles, grazing‐incidence diffraction packing motifs, or tip–SAM contact imaging) would be desirable to confirm the microscopic picture. However, difficulties related to the microscopic interpretation of those data mentioned below raise serious doubt of the achievability of this task.

We note that the orientation of alkanethiols adsorbed on metals has been extensively investigated by S K‐edge NEXAFS, photoelectron diffraction, grazing‐incidence X‐ray diffraction, and related techniques.[^14^, ^18^, ^64^, ^65^, ^66^, ^67^, ^68^, ^69^, ^70^, ^71^, ^72^, ^73^, ^74^, ^75^, ^76^] Despite this body of work, the microscopic configuration at the thiol adsorption site remains debated, and the great majority of structural data pertain to single‐crystal Au(111) rather than the polycrystalline electrodes employed here. Consequently we refrain from assigning the observed odd–even transport dichotomy to any specific adsorption geometry. The statistical analysis nevertheless establishes that odd and even members form two distinct classes with respect to both contact conductance and length attenuation.

## 3 CONCLUSION

Alkane monothiol CP‐AFM junctions have been the object of many studies by various groups in the past (e.g., Refs[^42^, ^48^, ^53^, ^60^, ^61^, ^62^, ^63^, ^77^, ^78^, ^79^, ^80^, ^81^]). While some thereof[^42^, ^48^, ^53^, ^60^, ^61^, ^62^, ^63^] reported the exponential falloff of the conductance with increasing size, they only interpreted this behavior as evidence for off‐resonant tunneling mechanism. Pooling all chain lengths regardless of parity, a possible odd‐even alternation was ruled out from the very beginning, and this created the impression that this effect only exists in large area EGaIn‐based junctions, where it was first observed.

The present study resolves longstanding debates on odd‐even effects in alkane monothiol junctions using CP‐AFM with Ag, Au, and Pt electrodes. Through rigorous statistical analysis of conductance G and molecule‐electrode coupling Γ, we demonstrate that:

- The effect is clearly present in CP‐AFM junctions once the data are analyzed with parity separation; earlier parity‐agnostic treatments concealed it.
- The relative conductance of odd versus even chains reverses with chain length (with the precise crossover subject to substantial uncertainty), thereby reconciling conflicting literature reports.
- Absolute odd–even differences are smaller on Ag than on Au because of exponential work‐function scaling, yet statistical confidence is highest for Ag owing to exceptional reproducibility of the even‐chain data.
- The effect arises from *both* interface (manifest in $G_{c}$ and $\Gamma_{c}$) and bulk (manifest in β and $\bar{\beta }$) contributions, although direct structural confirmation remains desirable.

This Solomonic solution provides a coherent framework for understanding odd‐even effects in molecular junctions and advances molecular electronics by clarifying quantum alternations at nanoscale interfaces. The parity‐dependent trade‐off between contact conductance and length decay offers a design principle: short odd chains maximize conductance, while long even chains are advantageous for length‐attenuated transport.

To end, from a more general perspective, along with the recent studies on the related n‐alk‐1‐yne (CnA) family[^82^, ^83^] this work draws attention to nontrivial effects of the molecular size on the charge transport in molecular junctions which cannot be captured by the ubiquitous single‐exponential behavior.

## 4 METHODS

Because our comprehensive characterization of CnT junctions and transport measurements were reported in detail in earlier studies,[^42^, ^49^, ^50^, ^51^] we only refer below to a few more significant details.

### 4.1 Materials

Gold nuggets (99.9% pure) were purchased from Mowrey, Inc. Silver pellets (99.99% pure) were purchased from Kurt J. Lesker Co. Evaporation boats and chromium evaporation rods were purchased from R. D. Mathis. Platinum and titanium for e‐beam evaporation were purchased from Kamis, Inc. Silicon (100) wafers were obtained from WaferNet. Contact mode AFM tips (DNP silicon nitride probes) were purchased from Bruker AFM Probes. 1‐heptanethiol (C7T, 98%), 1‐octanethiol (C8T, 98.5%), 1‐nonanethiol (C9T, 99%), 1‐decanethiol (C10T, 99%), 1‐undecanethiol (C11T, 98%), and 1‐dodecanethiol (C12T, 98%) were purchased from Sigma‐Aldrich Company.

### 4.2 Monolayer growth and characterization

Preparation of conducting tips and template‐stripped flat Ag, Au, and Pt substrates is described in a previous publication.^[48]^ SAMs were formed by immersing clean template‐stripped flat metal substrates into ethanol solutions of the molecules at a concentration of ∼1 mM for 20 h.

### 4.3 Transport measurements

The experimental setup for transport measurements was similar to that in our previous work.^[42]^ CP‐AFM‐based molecular junctions were constructed by mounting the substrates in the AFM and approaching the SAM with metal‐coated tips under ∼1 nN compressive load. Voltage was applied spanning ±1.5 V for CnT junctions. The slope of the low‐bias I–V characteristic (linear portion within ±0.1 V) was employed to extract low‐bias junction conductance G.

## CONFLICT OF INTEREST STATEMENT

The authors declare no conflicts of interest.

## ETHICS STATEMENT

No animal or human experiments were involved in this study.

## Supporting information

Supporting Information S1

## Data Availability

The data that support the findings of this study are available from the corresponding author upon reasonable request.
