# Supplementary material for: Odd‐even effects in molecular junctions: A Solomonic resolution of conflicting reports on conductance trends, metal specificity, and microscopic origin
Source: Smart Mol. 2026 Oct 8:e70108. Online ahead of print. doi: 10.1002/smo2.70108 (PMC13647442; doi:10.1002/smo2.70108)
Supplement: Supplementary file 1 [file SMO2-9999-0-s001.pdf]

Supporting Information for Odd-Even Effects in Molecular Junctions: A Solomonic Resolution of Conflicting Reports on Conductance Trends, Metal Specificity, and Microscopic Origin

Jiajun Feng,[†,‡] Ioan Bâldea,[*,¶] and Zuoti Xie[*,†,§]

†*Department of Materials Science and Engineering, Guangdong Provincial Key Laboratory of Materials and Technologies for Energy Conversion, Guangdong Technion-Israel Institute of Technology, Shantou, Guangdong, 515063, China*

‡*Schulich Faculty of Chemistry, Technion–Israel Institute of Technology, Haifa 3200003, Israel*

¶*Theoretical Chemistry, Heidelberg University, Im Neuenheimer Feld 229, D-69120 Heidelberg, Germany*

§*Quantum Science Center of Guangdong-Hong Kong-Macao Greater Bay Area (Guangdong), Shenzhen-Hong Kong International Science and Technology Park, NO.3 Binglang Road, Futian District, Shenzhen, Guangdong, 518000, China*

E-mail: ioan.baldea@pci.uni-heidelberg.de; zuoti.xie@gtiit.edu.cn

Single-Level Model Analysis of I–V Data

This section recalls the central expressions of the single-level model employed to process the I–V characteristics of the present junctions and explains the rationale for using the robust off-resonant form.

As a particular case of a general formula first deduced by Caroli et al,[1] the tunneling current through a single molecule junction mediated by a single level (MO, energy $E_{\mathrm{MO}} = E_F + \varepsilon_0$) electronically coupled to electrodes with wide flat conduction bands (Lorentzian transmission) can be expressed by an exact formula (Newns-Anderson model)

$$I = \frac{1}{e} G_0 \Gamma^2 \int_{-\infty}^{+\infty} dE \frac{f\left(E - E_F + eV/2\right) - f\left(E - E_F - eV/2\right)}{\left(E - E_F - \varepsilon_V\right)^2 + \Lambda^2} \tag{S1}$$

where $G_0 = 77.48\,\mu\mathrm{S}$ is the conductance quantum, $\Gamma = \sqrt{\Gamma_s \Gamma_t}$ is the effective coupling, $\varepsilon_V = \varepsilon_0 + \gamma eV$ is the bias-shifted MO energy, Λ is the HOMO broadening[2] (notice that typically $\Lambda \gg \Gamma$[2–4]), and $f(E)$ is the Fermi-Dirac function.

At low temperatures this reduces to[3,5]

$$I = G_0 \frac{\Gamma^2}{e\Lambda} \left[\arctan \frac{\varepsilon_V + eV/2}{\Lambda} - \arctan \frac{\varepsilon_V - eV/2}{\Lambda} \right]. \tag{S2}$$

In the off-resonant regime ($|\varepsilon_0| \gg \Lambda$ and $e|V| \lesssim 1.4|\varepsilon_0|$ as sampled here), the arctan expansion ($\arctan x \approx \pi/2 - 1/x$, accurate to better than 1% for $x > 10$) yields ($\varepsilon_h = -\varepsilon_0 > 0$ for HOMO-mediated conduction)[5]

$$I = G_0 \frac{\Gamma^2}{(-\varepsilon_h + \gamma eV)^2 - (eV/2)^2} V, \tag{S3}$$

with low-bias conductance

$$G = G_0 \frac{\Gamma^2}{\varepsilon_h^2} \tag{S4}$$

We apply the accurate eq. (S3) — and its low-bias limit, eq. (S4) — not only because it fits

the measured nonlinear I–V curves very well across the accessed bias range (Figure S2), but because it is microscopically justified by the assumption of Lorentzian transmission through a single dominant orbital (the HOMO). This assumption is directly validated experimentally: the HOMO offsets $\varepsilon_h^{\mathrm{TVS}}$ extracted from transition voltage spectroscopy (via the analytic $V_{t\pm}$ expressions derived from eq. (S3)) agree closely with independent UPS values $\varepsilon_h^{\mathrm{UPS}}$ (see Figure S1). Such agreement would not occur if the transmission deviated significantly from Lorentzian form. The near n-independence of ε_h further confirms that the HOMO energy of isolated alkanethiols is essentially constant, so the length dependence of G stems primarily from the exponential decay of Γ.

The transition voltages are obtained analytically from eq. (S3) as[5]

$$\gamma = -\frac{1}{2}\frac{V_{t+} + V_{t-}}{\sqrt{V_{t+}^2 + \frac{10|V_{t+}V_{t-}|}{3} + V_{t-}^2}} \tag{S5}$$

$$\varepsilon_h = 2\frac{e|V_{t+} + V_{t-}|}{\sqrt{V_{t+}^2 + \frac{10|V_{t+}V_{t-}|}{3} + V_{t-}^2}}. \tag{S6}$$

With ε_h determined this way (as a robust alternative to full I–V fitting) and the junction conductance $G_{\mathrm{junction}} = NG$ taken from the low-bias slope, the effective MO-electrode coupling Γ follows directly from eq. (S4). The Γ values used in the main analysis are listed in Table 1. Note that I and G in the above equations refer to a single-molecule junction. In CP-AFM measurements on CnT SAMs the picture of N parallel single-molecule junctions is valid,[4] so $G = G_{\mathrm{junction}}/N$ with $N = 80$ estimated from pull-off forces (Maugis-Dugdale contact mechanics[6]) and Rutherford backscattering/nuclear reaction analysis,[7] as detailed previously.[8]

Details on Statistical Tests for Parity Effects

In this supplementary section, we provide a detailed explanation of the statistical methods used to assess parity-dependent differences in fitting parameters (intercept $A = \ln G_c$ or

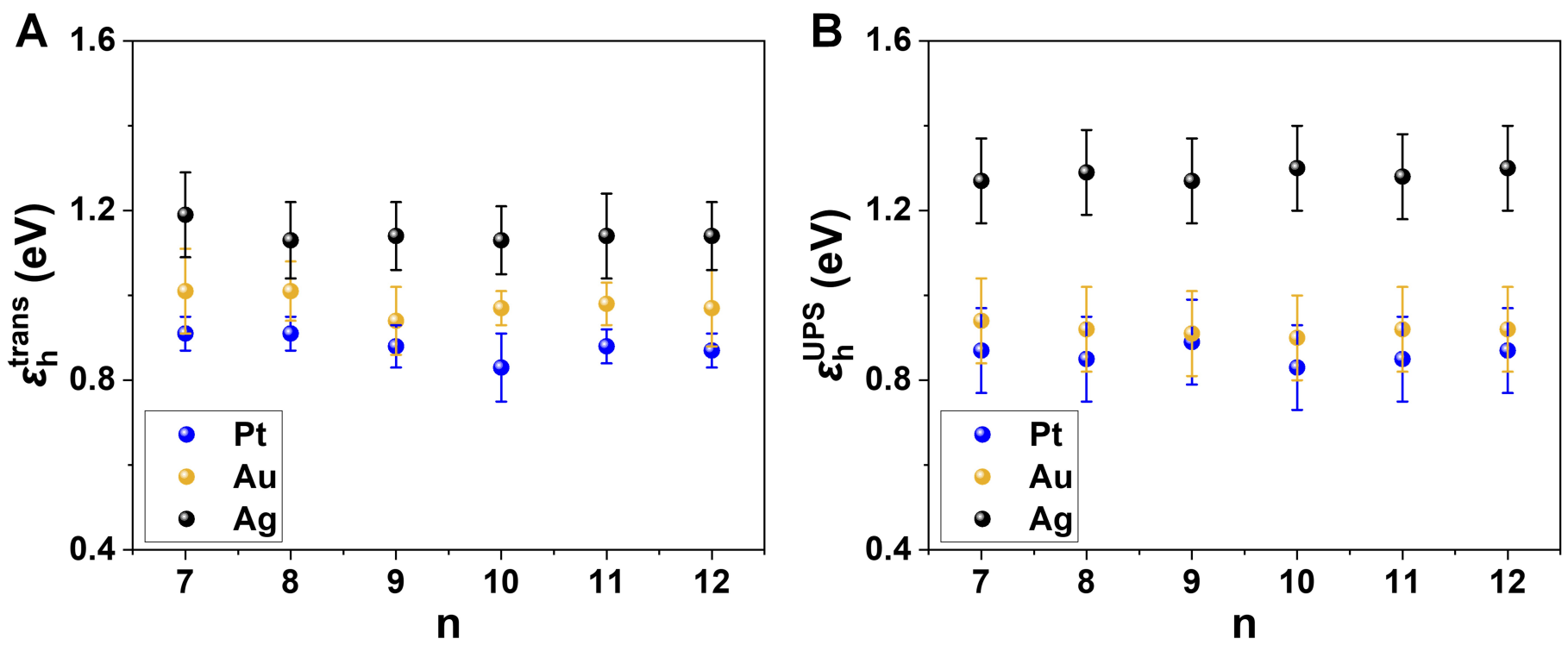

Figure S1: The agreement between the UPS-based values of the HOMO offset $\varepsilon_{h,UPS}$ values (left panel) and the TVS/transport-based ε_h data (right panel) for all sizes and metals confirm the Lorentzian-shape transmission, and hence the validity of the off-resonant single level model utilized.

$\ln \Gamma_c$, and slope β). The presentation is aimed at readers with an experimental background who are familiar with basic data analysis (e.g., computing averages and standard deviations in software like Origin) but may not routinely work with hypothesis testing. We begin with intuitive explanations using everyday analogies, followed by mathematical definitions.

Intuitive Explanation of Key Concepts

Statistical tests help answer a simple question: "Is the difference we see between two groups (e.g., odd- and even-parity chains) likely due to a real physical effect, or could it just be random scatter in the measurements?" Think of it like repeating an experiment several times and getting slightly different results each time due to noise — the test quantifies how surprising the observed difference would be if there were no underlying effect.

The p-value: How surprising is the result under "no effect"?

The p-value is the probability of seeing a difference at least as large as the one in your actual data (or larger) if there truly were no real difference between the groups — i.e., if the odd and even chains behaved identically, and all variation was just random noise.

- **Everyday analogy:** Imagine flipping a fair coin 20 times and getting 15 heads. If

the coin is fair (50/50 chance), the p-value answers: "How likely is it to get 15 or more heads (or 5 or fewer) just by chance?" A small p-value (e.g., $p = 0.01$) means it's unlikely — like winning a small lottery — suggesting the coin might be biased. If one got exactly 10 heads, p would be high (not surprising), so no evidence of bias.

- **Lab-friendly version:** Suppose one measures the conductance of two samples and gets averages that differ by 20%, but with standard deviations of 10%. The p-value tells us: "If the samples were actually identical, how often would random scatter produce a difference this big or bigger?" If $p < 0.05$ (less than 5% chance), it's conventionally called "significant" — probably not just noise. In our analysis, small p-values (e.g., 8.5×10^{-4} for β on Ag) indicate strong evidence for a parity (odd-even alternation) effect.

Common thresholds:

- $p < 0.001$: Very convincing ("highly significant")
- $p = 0.01 - 0.05$: Suggestive ("significant")
- $p = 0.05 - 0.15$: A trend worth noting, but needs more data

Think of p as the "false alarm" rate — how often one would cry "discovery!" when it's just noise.

The z-score: How many "noise levels" away is the difference?

The z-score measures the size of the observed difference relative to the typical variability (uncertainty) in the measurements. It's like asking: "How many standard deviations is this result from what you'd expect if nothing interesting was happening?"

- **Everyday analogy:** If average height in a group is 170 cm with a standard deviation of 10 cm, a person at 200 cm is 3 standard deviations ($z = 3$) above average — unusually tall. $z = 1$ (180 cm) is common; $z = 5$ (220 cm) is extraordinary.

- **Lab-friendly version:** For our fits, the z-score for the prefactor $A = \ln G_c$ on Ag is 4.11 (Table 2 of the main text), meaning the difference between odd and even prefactors is over 4 times larger than the combined uncertainty. Larger $|z|$ means stronger evidence ($z > 2$ is often noteworthy; $z > 3$ is convincing). If error bars (standard errors) don't overlap much, z will be large.

One-sided vs. two-sided tests: Direction matters

- **Two-sided test:** Checks for any difference, regardless of direction (e.g., "Are odd and even different at all?"). It considers surprises on both sides (odd higher or lower than even). Analogy: Testing if a new catalyst changes reaction rate (faster or slower).

- **One-sided test:** Checks only in the expected direction (e.g., "Is odd greater than even?", based on physical intuition or prior data). This is more sensitive if there is a good reason to expect one direction — it roughly halves the p-value compared to two-sided. Analogy: Testing if the catalyst makes the reaction faster (one does not care if it is slower).

In this work, one-sided tests are employed because the measured data consistently show larger A and β for odd-parity chains across all investigated metals. This "Solomonic" resolution — higher short-chain conductance but faster decay for odd chains — aligns with the observed data and motivates directional testing. Importantly, the more conservative two-sided tests do not alter the main conclusions. For the Ag prefactor and decay constant the two-sided p-values remain 3.9×10^{-5} and 1.7×10^{-3}, respectively, still corresponding to confidence levels $> 99.8\,\%$. Thus the statistical robustness of the odd–even effect on Ag is independent of the one- versus two-sided choice.

Welch's t-Test: Handling Unequal Scatter

This variant of the common t-test does not assume the two groups have the same amount of variability (variance). It's like comparing averages from two experiments where one has

more scatter than the other — precisely our situation with odd and even members.

- **Everyday analogy:** Comparing heights of men and women, but men's heights vary more (larger standard deviation). A standard t-test might overestimate confidence; Welch's test adjusts for that.
- **Lab-friendly version:** In our data, odd-parity fits often have larger standard errors than even-parity fits (due to more variability in measurements). Welch's test accounts for this, making the comparison fairer. If variances were equal, it reduces to the standard t-test.

Welch's t-Test and Combined Uncertainty

When comparing two independent estimates $\hat{\theta}_1$ and $\hat{\theta}_2$ (e.g., prefactors for odd and even chains) with their respective standard errors s_1 and s_2, the uncertainty of the difference is not simply the sum or average of the individual uncertainties. Because the two estimates come from independent measurements, their errors are uncorrelated, and the variance of the difference is the *sum* of the individual variances

$$\mathrm{Var}(\hat{\theta}_1 - \hat{\theta}_2) = s_1^2 + s_2^2 \tag{S7}$$

The *combined standard error* is the square root of this variance

$$\sigma_{\mathrm{diff}} = \sqrt{s_1^2 + s_2^2} \tag{S8}$$

This forms the denominator in the z-score defined as

$$z = \frac{\hat{\theta}_1 - \hat{\theta}_2}{\sqrt{s_1^2 + s_2^2}} \tag{S9}$$

Intuitive analogy: Imagine measuring the heights of two groups with rulers that have different precisions. The uncertainty in the *difference* between the average heights is larger

than either individual uncertainty, but smaller than their sum. The formula $\sqrt{s_1^2 + s_2^2}$ correctly combines them—like the Pythagorean theorem for uncertainties.

For Ag prefactors: $s_o = 0.34$, $s_e = 0.04$, so $\sigma_{\text{diff}} = \sqrt{0.34^2 + 0.04^2} = \sqrt{0.1156 + 0.0016} = \sqrt{0.1172} = 0.342$. The difference $\ln G_{c,o} - \ln G_{c,e} = (-9.53) - (-10.92) = 1.39$, yielding $z = 1.39/0.342 = 4.06$, consistent with the $z = 4.11$ in the main text (minor rounding differences).

For the full Welch's t-test, which accounts for potentially different sample sizes, the test statistic is

$$t = \frac{\hat{\theta}_1 - \hat{\theta}_2}{\sqrt{\frac{s_1^2}{n_1} + \frac{s_2^2}{n_2}}} \tag{S10}$$

where n_1 and n_2 are the number of data points used to estimate $\hat{\theta}_1$ and $\hat{\theta}_2$, respectively. In our case, for each metal and parity, we have three chain lengths ($n = 7, 9, 11$ for odd; $n = 8, 10, 12$ for even), so $n_1 = n_2 = 3$. Because the sample sizes are equal and small, the z-score approximation (which uses $s_1^2 + s_2^2$ in the denominator) differs slightly from the full Welch's t-statistic (which uses $s_1^2/n_1 + s_2^2/n_2$). However, for our purposes, the z-score provides a clear and conservative estimate of statistical significance.

The degrees of freedom ν for Welch's t-test are approximated as

$$\nu \approx \frac{\left(\frac{s_1^2}{n_1} + \frac{s_2^2}{n_2}\right)^2}{\frac{s_1^4}{n_1^2(n_1-1)} + \frac{s_2^4}{n_2^2(n_2-1)}} \tag{S11}$$

For our data ($n_1 = n_2 = 3$), ν is typically small (around $2-4$), meaning the t-distribution has heavier tails than the normal distribution. However, because our z-scores are large (e.g., 4.11 for Ag), the conclusions remain robust regardless of whether we use the normal or t-distribution.

Normal Distribution and Cumulative Distribution Function $\Phi(z)$

The standard normal distribution has probability density function:

$$\phi(z) = \frac{1}{\sqrt{2\pi}} \exp\left(-\frac{z^2}{2}\right)$$

The cumulative distribution function $\Phi(z)$ gives the probability that a random variable from this distribution is less than or equal to z:

$$\Phi(z) = \int_{-\infty}^{z} \phi(t)\, dt = \frac{1}{2}\left[1 + \text{erf}\left(\frac{z}{\sqrt{2}}\right)\right]$$

where $\text{erf}(x) = \frac{2}{\sqrt{\pi}} \int_0^x \exp(-t^2)\, dt$ is the error function (available in most scientific software).

For positive z, $\Phi(z)$ is the confidence level (as a fraction) that $\theta_1 > \theta_2$. We report it as a percentage, e.g., $\Phi(1.96) \approx 0.975$ or 97.5%.

***p*-value**

For a one-sided test ($\hat{\theta}_1 > \hat{\theta}_2$):

$$p = 1 - \Phi(z) = \frac{1}{2}\left[1 - \text{erf}\left(\frac{z}{\sqrt{2}}\right)\right]$$

For two-sided tests, it's $2(1 - \Phi(|z|))$. Small p means reject the "no difference" hypothesis.

A confidence level exceeding 95% (corresponding to $p < 0.05$ for two-sided tests) is conventionally interpreted as strong evidence against the null hypothesis (i.e, the effect is real). However, to allow nuanced interpretation, we report exact confidence percentages, z-scores, and p-values in the corresponding tables.

This statistical framework is more reliable than merely comparing best-fit values with their individual standard deviations, because it properly accounts for the combined uncertainty of both estimates. Many experimental studies overlook this step and risk overinterpreting small differences that may be consistent with random fluctuations. In the present

work, computed z-scores and confidence levels provide quantitative evidence that the odd-even effect is statistically real.

The parameter distributions shown in the main-text figures are constructed as normal distributions $\mathcal{N}(\hat{\theta}, [\mathrm{se}(\hat{\theta})]^2)$ centered on the least-squares estimates. They therefore illustrate the parametric uncertainty that enters the z-score (or Welch) calculation. Nonparametric bootstrap resampling of the underlying I–V traces was not performed because the primary experimental observables already enter the analysis as averaged conductances and extracted Γ values; the relevant uncertainty is that of the subsequent linear-regression parameters, which is correctly captured by the reported standard errors under the usual normality assumptions of ordinary least squares. For the Ag even-parity series the residuals are so small that any bootstrap distribution would be essentially indistinguishable from the parametric normal used here.

Monte Carlo Simulation for Crossover Length Uncertainty

The crossover length defined in eq. (8) is a ratio of fitted parameters:

$$n_{\text{cross}} = \frac{\ln \overline{G}_{c,e} - \ln \overline{G}_{c,o}}{\beta_o - \beta_e}$$

Because even small uncertainties in the denominator $\beta_o - \beta_e$ are amplified in such ratios, quantitative estimates of n_{cross} must be interpreted with caution. We performed Monte Carlo simulations to assess the reliability of these estimates and to illustrate why the main text adopts a cautious stance.

The following Python code illustrates the procedure for Ag conductance data, using a fixed random seed for reproducibility:

```
import numpy as np

from scipy.stats import norm
```

```
# --- Input: parameter estimates and standard errors from fit (Ag conductance) ---
# From Table 2 (Model 3) in the main text
A_odd     = -9.5337
se_A_odd  =  0.3351
beta_odd  =  1.2317
se_beta_odd = 0.0366

A_even     = -10.9228
se_A_even  =  0.0413
beta_even  =  1.1161
se_beta_even = 0.0041

# Number of Monte Carlo samples
n_samples = 100000
np.random.seed(42)  # fixed seed for reproducibility

# --- Monte Carlo sampling ---
# Draw random samples from normal distributions
A_odd_samples     = norm.rvs(loc=A_odd,     scale=se_A_odd,     size=n_samples)
A_even_samples    = norm.rvs(loc=A_even,    scale=se_A_even,    size=n_samples)
beta_odd_samples  = norm.rvs(loc=beta_odd,  scale=se_beta_odd,  size=n_samples)
beta_even_samples = norm.rvs(loc=beta_even, scale=se_beta_even, size=n_samples)

# Compute n_cross for each sample using the formula from eq. (14)
# n_cross = (ln G_{c,e} - ln G_{c,o}) / (beta_o - beta_e)
# To obtain positive values directly, we use denominator = beta_even - beta_odd
denom = beta_even_samples - beta_odd_samples  # negative
```

```
n_cross = (A_even_samples - A_odd_samples) / denom  # negative/negative = positive

# Filter out samples where denominator is too close to zero
threshold = 1e-10
valid = np.abs(denom) > threshold
n_cross_valid = n_cross[valid]

# --- Summary statistics ---
if len(n_cross_valid) > 0:
    median_n_cross = np.median(n_cross_valid)
    ci_low  = np.percentile(n_cross_valid, 2.5)
    ci_high = np.percentile(n_cross_valid, 97.5)

    print(f"Median n_cross: {median_n_cross:.2f}")
    print(f"95% Monte Carlo CI: [{ci_low:.1f}, {ci_high:.1f}]")
    print(f"Fraction valid: {len(n_cross_valid)/n_samples:.3f}")
```

Running this code yields the following output (reproducible due to the fixed seed):

```
Median n_cross: 12.01
95% Monte Carlo CI: [5.4, 33.6]
Fraction valid: 1.000
```

The relatively wide confidence interval ([5.4, 33.6]) reflects the sensitivity of the ratio to small differences in β. Although all samples yielded physically meaningful denominators (fraction valid = 1.000), the uncertainty in $\beta_o - \beta_e$ propagates to substantial uncertainty in n_{cross}. This supports the cautious stance taken in the main text: while the existence of a crossover is robust, its precise numerical value cannot be reliably determined from the

present data (n = 7–12). Extending measurements to longer chains would substantially reduce this uncertainty.

Key points:

- Parameters are sampled independently from $\mathcal{N}(\hat{\theta}, [\mathrm{se}(\hat{\theta})]^2)$.
- The fixed seed (42) ensures that readers can reproduce exactly these numbers.
- Samples with denominator $|\beta_{\text{even}} - \beta_{\text{odd}}|$ extremely close to zero are discarded as unphysical, though none occurred in this simulation.
- The resulting n_{cross} distribution is asymmetric, explaining why confidence intervals are reported rather than simple standard errors.
- Increasing samples to 100,000 improves precision of percentiles but does not change the qualitative conclusion.

This Monte Carlo approach makes no strong assumptions about the n_{cross} distribution shape and directly reflects propagated uncertainty from linear regression fits. It is particularly useful when extrapolating beyond the measured range (n = 7–12), as done here.

Additional Tables for Conductance Fitting

Table S1: **Parity-Independent Fitting of Conductance G.** Best-fit parameters and coefficient of determination R^2 for models assuming no odd-even alternation. **Top panel:** Metal-dependent fits to eq. (1) ($G = G_c \exp(-\beta n)$). **Bottom panel:** Global fit to eq. (2) ($G = \overline{G}_c \exp(-\beta n + \xi\Phi)$) with metal-independent parameters.

Metal	$\ln G_c$	β	R^2
Ag	-9.96 ± 0.49	1.20 ± 0.05	0.9929
Au	-7.56 ± 0.44	1.18 ± 0.05	0.9942
Pt	-5.49 ± 0.47	1.20 ± 0.05	0.9934

$\ln \overline{G}_c$	β	ξ (eV^{-1})	R^2
-23.44 ± 0.78	1.193 ± 0.044	$+3.134 \pm 0.130$	0.9887

Table S2: **Parity-dependent fitting results and statistical significance for conductance G using eq. (5)** ($G_p = \overline{G}_{c,p} \exp(-\beta_p n + \xi_p \Phi)$). Top panel: metal- and parity-dependent best-fit parameters ($\ln G_{c,p}$, β_p, ξ_p, R^2). The metal-independent fitting parameters only depend on parity. Bottom panel: confidence levels (%), z-scores and p-values testing whether odd chains have larger G-related properties than even chains.

Parity	$\ln \overline{G}_{c,p}$	β	ξ (eV^{-1})	R^2
Odd	-23.02 ± 1.11	1.218 ± 0.066	$+3.119 \pm 0.185$	0.9905
Even	-24.35 ± 1.03	1.122 ± 0.059	$+3.148 \pm 0.166$	0.9917

Parameter	Confidence (%)	z-score	p-value
$\ln \overline{G}_{c,o} > \ln \overline{G}_{c,e}$	80.9	+0.88	0.191
$\beta_o > \beta_e$	86.1	+1.08	0.139
$\xi_o > \xi_e$	45.4	-0.12	0.546

Table S3: **Parity-dependent fitting results and statistical significance for conductance G using eq. (6)** ($G_p = \overline{G}_p \exp(-\beta_p n + \xi \Phi)$). Top panel: metal- and parity-dependent best-fit parameters. The fitting assumes parity-dependent $\ln \overline{G}_{c,p}$ and β_p and global $\xi = 3.134 \pm 0.119$ eV^{-1}.. Bottom panel: confidence levels (%), z-scores and p-values testing whether odd chains have larger G-related properties than even chains.

Parity	$\ln \overline{G}_c$	β	R^2
Odd	-23.09 ± 0.82	1.218 ± 0.060	0.9905
Even	-24.28 ± 0.86	1.122 ± 0.060	0.9917

Parameter	Confidence (%)	z-score	p-value
$\ln \overline{G}_{c,o} > \ln \overline{G}_{c,e}$	84.1	+1.00	0.159
$\beta_o > \beta_e$	87.0	+1.13	0.130

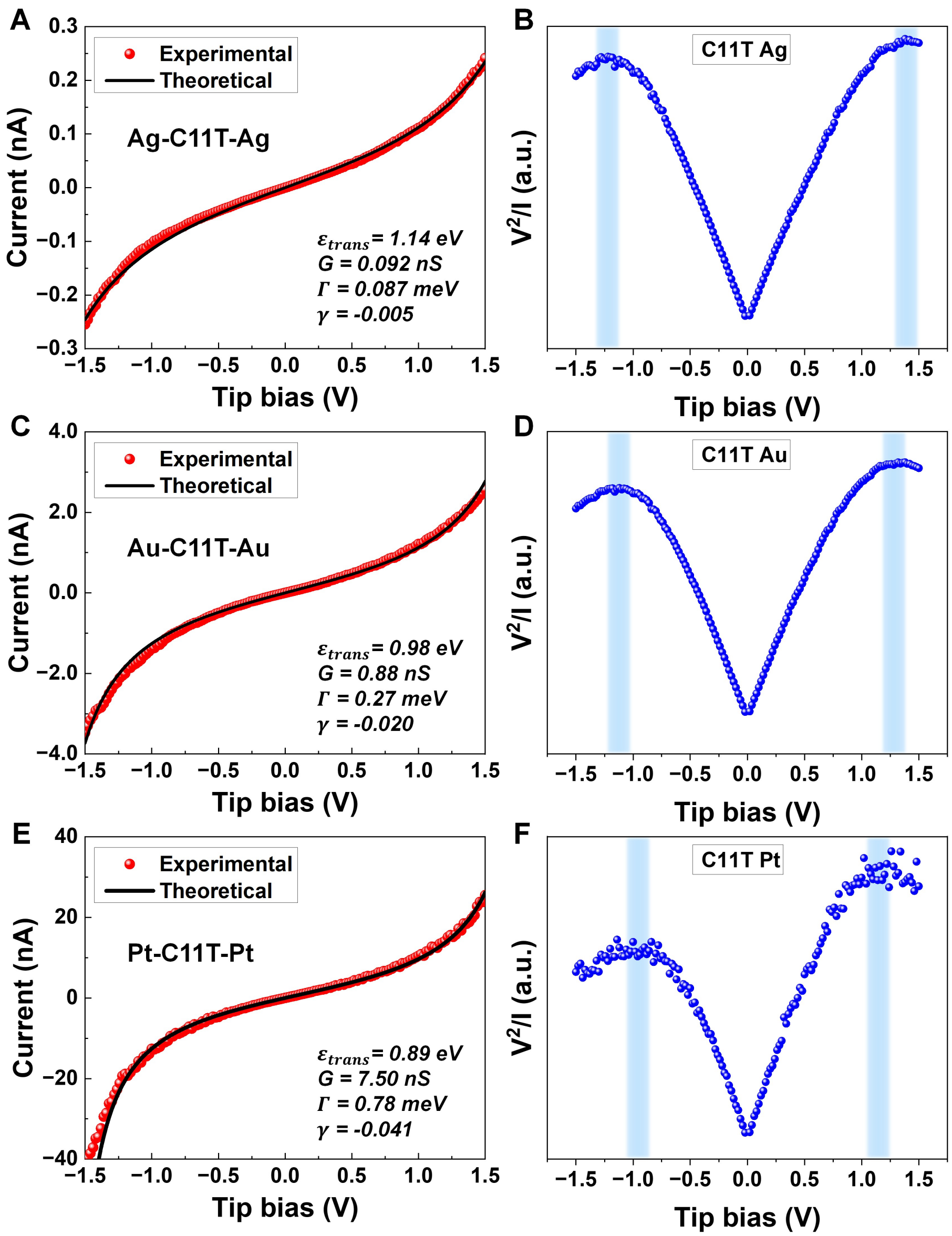

Figure S2: (A,C,E) Representative individual raw I–V traces measured on CP-AFM C11T junctions with Ag, Au, and Pt electrodes, overlaid with fits using the off-resonant single-level expression (eq. (S3)) (B,D,F) recast as TVS plots $V^2/|I|$ versus V.

Models and Additional Tables for the HOMO-Electrode Coupling Γ

$$\Gamma = \Gamma_c \exp(-\overline{\beta} n), \ln\Gamma = \ln\Gamma_c - \overline{\beta} n \tag{S12}$$

$$\Gamma = \overline{\Gamma}_c \exp(-\overline{\beta} n + \overline{\xi}\Phi), \ln\Gamma = \ln\Gamma_c - \overline{\beta} n + \overline{\xi}\Phi \tag{S13}$$

$$\Gamma_p = \Gamma_{c,p} \exp\left(-\overline{\beta}_p n\right), \ln\Gamma_p = \ln\Gamma_{c,p} - \overline{\beta}_p n \tag{S14}$$

$$\Gamma_{n,p} = \overline{\Gamma}_p \exp\left(-n\overline{\beta}_{p,m} + \overline{\xi}_p\Phi\right), \ln\Gamma_{n,p} = \ln\overline{\Gamma}_p - n\overline{\beta}_{p,m} + \overline{\xi}_p\Phi \tag{S15}$$

$$\Gamma_{n,p} = \overline{\Gamma}_p \exp\left(-n\overline{\beta}_p + \overline{\xi}_p\Phi\right), \ln\Gamma_{n,p} = \ln\overline{\Gamma}_p - n\overline{\beta}_p + \overline{\xi}_p\Phi \tag{S16}$$

$$\Gamma_{n,p} = \overline{\Gamma}_p \exp\left(-n\overline{\beta}_p + \overline{\xi}\Phi\right), \ln\Gamma_{n,p} = \ln\overline{\Gamma}_p - n\overline{\beta}_p + \overline{\xi}\Phi \tag{S17}$$

Table S4: **Parity-Independent Fitting of the MO-Electrode Coupling Γ.** Best-fit parameters and coefficient of determination R^2 for models assuming no odd-even alternation. **Top panel:** Metal-dependent fits to eq. (S12) ($\Gamma = \Gamma_c \exp(-\overline{\beta} n)$). **Bottom panel:** Global fit to eq. (S13) ($\Gamma = \overline{\Gamma}_c \exp(-\overline{\beta} n + \overline{\xi}\Phi)$) with metal-independent parameters.

Metal	$\ln\Gamma_c$	$\overline{\beta}$	R^2
Ag	+4.66 ± 0.28	0.61 ± 0.03	0.9906
Au	+5.71 ± 0.23	0.60 ± 0.02	0.9934
Pt	+6.68 ± 0.25	0.61 ± 0.03	0.9927

$\ln\overline{\Gamma}_c$	$\overline{\beta}$	$\overline{\xi}$ (eV^{-1})	R^2
-1.28 ± 0.38	0.604 ± 0.022	1.383 ± 0.063	0.9883

Data Fitting to Parity-Agnostic Models

The results of data fitting to these models — eqs. (1) and (2) for G and their mirrors for Γ (eqs. (S12) and (S13)) — are depicted in Figures S3 and S4.

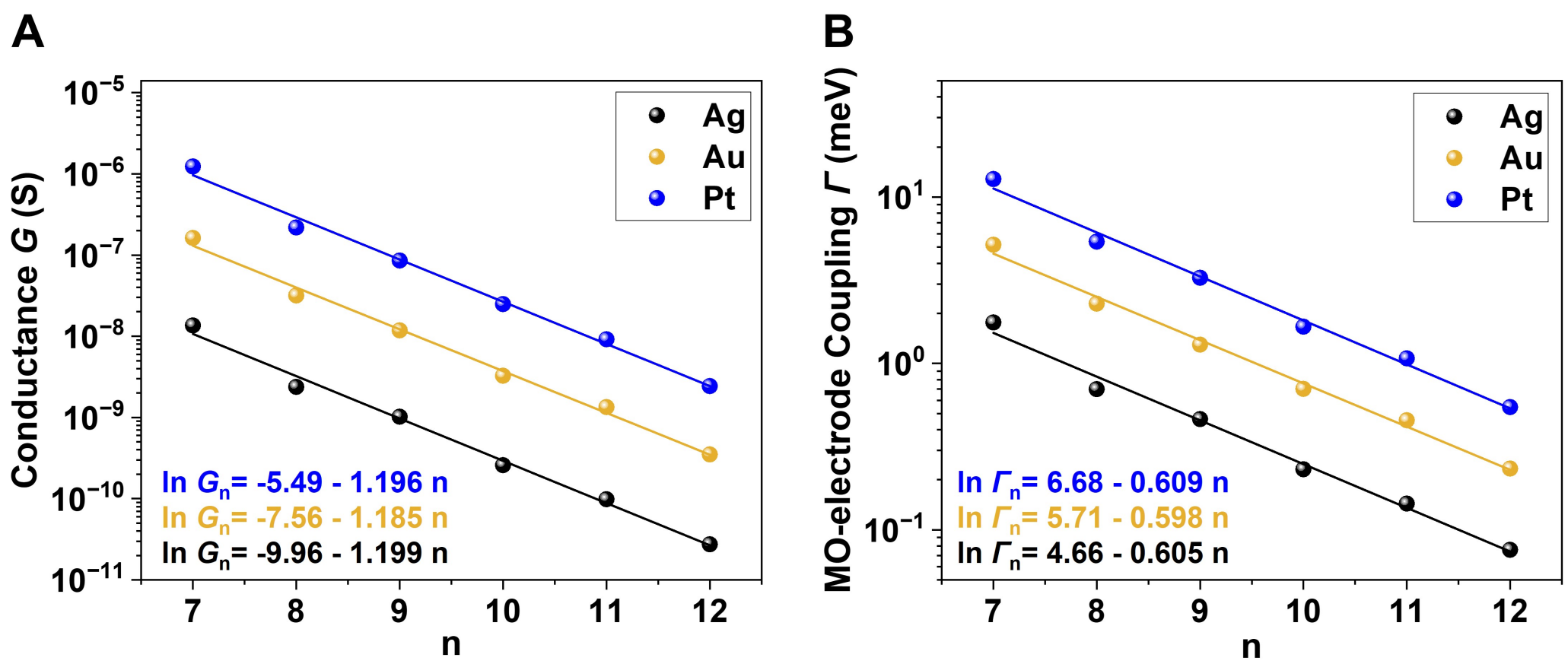

Figure S3: Parity-agnostic fits assuming no odd-even alternation. (A) Conductance G fitted to eq. (1): (B) HOMO-electrode coupling Γ fitted to eq. (S12). Data for all chain lengths ($n =$ 7–12) are pooled regardless of parity. Prefactors (G_c, Γ_c) and decay coefficients (β, $\overline{\beta}$) are metal-dependent; numerical values are given in Tables S1 and S4. These fits, while capturing the overall exponential decay, conceal the odd-even alternation that becomes evident upon parity separation (cf. Figures 2–4).

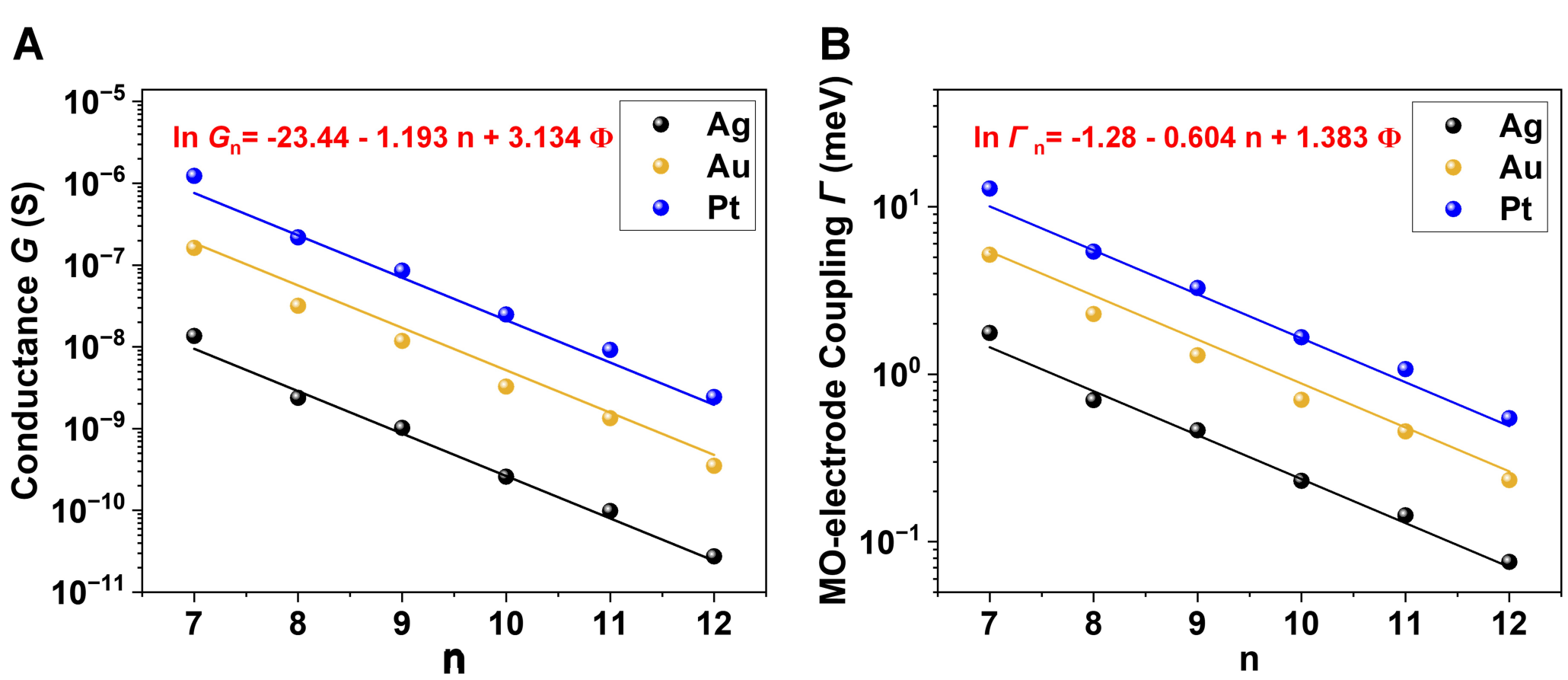

Figure S4: Parity-agnostic global fit incorporating work-function dependence. (A) Conductance G fitted to eq. (2); (B) HOMO-electrode coupling Γ fitted to eq. (S13). Parameters ($\overline{G}_c$, $\overline{\Gamma}_c$, β, $\overline{\beta}$, ξ, $\overline{\xi}$) are metal-independent, assuming no odd-even alternation. While the work-function scaling ($\xi \approx 3.1$ eV^{-1}) captures the metal dependence well, parity-specific effects remain hidden. Numerical values are given in Tables S1 and S4.

Table S5: **Parity-dependent fitting results and statistical significance for Γ using eq. (S16) ($\Gamma_{n,p} = \overline{\Gamma}_p \exp\left(-n\overline{\beta}_p + \overline{\xi}_p \Phi\right)$)** Top panel: metal- and parity-dependent best-fit parameters ($\ln \Gamma_{c,p}$, β_p, ξ_p, R^2). The metal-independent fitting parameters only depend on parity. Bottom panel: confidence levels (%), z-scores and p-values testing whether odd chains have larger Γ-related properties than even chains.

Parity	$\ln \overline{\Gamma}_c$	$\overline{\beta}$	$\overline{\xi}$ (eV^{-1})	R^2
Odd	-1.05 ± 0.57	0.618 ± 0.034	$+1.374 \pm 0.095$	0.9891
Even	-1.76 ± 0.44	0.566 ± 0.025	$+1.391 \pm 0.070$	0.9933

Parameter	Confidence (%)	z-score	p-value
$\ln \overline{\Gamma}_{c,o} > \ln \overline{\Gamma}_{c,e}$	84.1	+1.00	0.159
$\overline{\beta}_o > \overline{\beta}_e$	88.9	+1.22	0.111
$\overline{\xi}_o > \overline{\xi}_e$	44.1	-0.15	0.559

Table S6: **Parity-dependent fitting results and statistical significance for Γ using eq. (S17) ($\Gamma_{n,p} = \overline{\Gamma}_p \exp\left(-n\overline{\beta}_p + \overline{\xi}\Phi\right)$).** Top panel: metal- and parity-dependent best-fit parameters. The fitting assumes parity-dependent $\ln \overline{\Gamma}_{c,p}$ and $\overline{\beta}_p$ and global $\overline{\xi} = 1.383 \pm 0.063$ eV^{-1}. Bottom panel: confidence levels (%), z-scores and p-values testing whether odd chains have larger Γ-related properties than even chains.

Parity	$\ln \overline{\Gamma}_{c,p}$	$\overline{\beta}_p$	R^2
Odd	-1.09 ± 0.39	0.618 ± 0.029	0.9891
Even	-1.72 ± 0.41	0.566 ± 0.029	0.9933

Parameter	Confidence (%)	z-score	p-value
$\ln \overline{\Gamma}_{c,o} > \ln \overline{\Gamma}_{c,e}$	86.8	+1.12	0.132
$\overline{\beta}_o > \overline{\beta}_e$	89.8	+1.27	0.102

Mechanistic Interpretation

To obtain a quantitative estimate of the work-function coefficient ($\xi \approx 3.1\,\mathrm{eV}^{-1}$) is a difficult task, but the fact that it is parity independent can be understood. It follows from the parity independence of the relevant properties related to it.

Using state-of-the-art ab initio quantum chemical calculations, we showed in the past that the HOMO energy of the isolated CnT molecules (E^0_{HOMO} does not depend on the size (hence, they are parity insensitive).[9] A quantitative estimate of the image-driven HOMO shift for CnT junctions is difficult. Considering a point-like HOMO is an inadequate description.[10] Convoluting ($\int d^3\mathbf{r}|\Psi(\mathbf{r})|^2 V(z)$) the realistic HOMO spatial distribution (described in terms

of its wave function Ψ) with the image charge potential $V(z)$ which is needed to estimate the image-driven HOMO shift is problematic.

CnT's HOMO spatial distribution is mainly concentrated near the thiol end, close to the substrate ($z \to 0$), where the classical image potential $V(z)$ diverges as $V(z) \propto 1/(4z)$. A quantitative estimate therefore requires an adequate cutoff (e.g., ref. 10 and citations therein), and the result depends on the chosen cutoff. However, because the HOMO distribution is parity-independent, any image-driven shift affects odd and even chains equally regardless of the cutoff details.

A similar argument holds for the interface dipole effect. Following an argument verified for the related n-alk-1-yne series (CnA),[11,12] the tilt (polar) angle $\angle(\mathrm{Au}, \mathrm{S}, \mathrm{C})$ is dictated by the local chemistry at the thiol end;[11] it is independent of the (size and hence of the) parity. This makes the HOMO-shift due to interface dipoles also parity independent. If the aforementioned are correct, the HOMO energy offset of the CnT molecules embedded in CP-AFM junctions ($\varepsilon_h = E_F - E_{\mathrm{HOMO}}$ is (size and hence) parity independent. In this way, we arrived to a conclusion whose correctness is substantiated by the values presented in Table 1: across all sizes ε_h remains essentially independent of n for each metal (maximum variation 0.07 eV).

To sum up, the statistical indistinguishability of ξ (and $\overline{\xi}$) for odd and even chains reinforces the conclusion that the parity effect originates in structural/contact factors rather than in a parity-dependent modification of interfacial electrostatics.

References

(1) Caroli, C.; Combescot, R.; Nozieres, P.; Saint-James, D. Direct Calculation of the Tunneling Current. *J. Phys. C: Solid State Phys.* **1971**, *4*, 916.

(2) Bâldea, I. Dichotomy between Level Broadening and Level Coupling to Electrodes in Large Area EGaIn Molecular Junctions. *J. Phys. Chem. Lett.* **2024**, *15*, 2916–2921.

(3) Bâldea, I. Can Tunneling Current in Molecular Junctions Be So Strongly Temperature Dependent to Challenge a Hopping Mechanism? Analytical Formulas Answer This Question and Provide Important Insight into Large Area Junctions. *Phys. Chem. Chem. Phys.* **2024**, *26*, 6540–6556.

(4) Chen, Y.; Feng, J.; Bâldea, I.; Frisbie, C. D.; Xie, Z. Intermolecular Interactions in Molecular Tunnel Junctions: Significant and Still Compatible with the Picture of Single Molecule Junctions in Parallel. *JACS Au* **2025**, *5*, 4995–5005.

(5) Bâldea, I. Ambipolar Transition Voltage Spectroscopy: Analytical Results and Experimental Agreement. *Phys. Rev. B* **2012**, *85*, 035442.

(6) Maugis, D. Adhesion of Spheres: The JKR-DMT Transition Using a Dugdale Model. *J. Colloid. Interf. Sci.* **1992**, *150*, 243 – 269.

(7) Demissie, A. T.; Haugstad, G.; Frisbie, C. D. Quantitative Surface Coverage Measurements of Self-Assembled Monolayers by Nuclear Reaction Analysis of Carbon-12. *J. Phys. Chem. Lett.* **2016**, *7*, 3477–3481.

(8) Xie, Z.; Bâldea, I.; Demissie, A. T.; Smith, C. E.; Wu, Y.; Haugstad, G.; Frisbie, C. D. Exceptionally Small Statistical Variations in the Transport Properties of Metal-Molecule-Metal Junctions Composed of 80 Oligophenylene Dithiol Molecules. *J. Am. Chem. Soc.* **2017**, *139*, 5696–5699.

(9) Xie, Z.; Bâldea, I.; Frisbie, C. D. Energy Level Alignment in Molecular Tunnel Junctions by Transport and Spectroscopy: Self-Consistency for the Case of Alkyl Thiols and Dithiols on Ag, Au, and Pt Electrodes. *J. Am. Chem. Soc.* **2019**, *141*, 18182–18192.

(10) Bâldea, I. Transition Voltage Spectroscopy Reveals Significant Solvent Effects on Molecular Transport and Settles an Important Issue in Bipyridine-Based Junctions. *Nanoscale* **2013**, *5*, 9222–9230.

(11) Chen, Y.; Bâldea, I.; Koren, E.; Xie, Z. Conformational Bifurcation Drives Dual Transport Regimes in Molecular Junctions: Unsupervised Machine Learning Insights. *Smart Molecules* *4*, e70033.

(12) Bâldea, I. Unlocking n-alk-1-ynes conformers: Quantum "Trigger Finger" versus "Stiff Joint" Conformations. *Smart Molecules* **2026**, *doi https://doi.org/10.1002/smo2.70054*, e70054.
